# Blood-based DNA methylation in advanced Nasopharyngeal Carcinoma exhibited distinct CpG methylation signature

**DOI:** 10.1038/s41598-023-45001-w

**Published:** 2023-12-12

**Authors:** Koustav Chatterjee, Sudipa Mal, Monalisha Ghosh, Nabanita Roy Chattopadhyay, Sankar Deb Roy, Koushik Chakraborty, Syamantak Mukherjee, Moatoshi Aier, Tathagata Choudhuri

**Affiliations:** 1 Department of Biotechnology, Visva-Bharati, Santiniketan, Birbhum, West Bengal India 731235; 2 Department of Radiation Oncology, Eden Medical Center, Dimapur, Nagaland India; 3 Department of Pathology, Eden Medical Center, Dimapur, Nagaland India

**Keywords:** Cancer, Head and neck cancer

## Abstract

The TNM staging system is currently used to detect cancer stages. Regardless, a small proportion of cancer patients recur even after therapy, suggesting more specific molecular tools are required to justify the stage-specific detection and prompt cancer diagnosis. Thus, we aimed to explore the blood-based DNA methylation signature of metastatic nasopharyngeal carcinoma (NPC) to establish a holistic methylation biomarker panel. For the identification of methylation signature, the EPIC BeadChip-based array was performed. Comparative analysis for identifying unique probes, validation, and functional studies was investigated by analyzing GEO and TCGA datasets. We observed 4093 differentially methylated probes (DMPs), 1232 hydroxymethylated probes, and 25 CpG islands. Gene expression study revealed both upregulated and downregulated genes. Correlation analysis suggested a positive (with a positive r, p ≤ 0.05) and negative (with a negative r, p ≤ 0.05) association with different cancers. TFBS analysis exhibited the binding site for many TFs. Furthermore, gene enrichment analysis indicated the involvement of those identified genes in biological pathways. However, blood-based DNA methylation data uncovered a distinct DNA methylation pattern, which might have an additive role in NPC progression by altering the TFs binding. Moreover, based on tissue-specificity, a variation of correlation between methylation and gene expression was noted in different cancers.

## Introduction

Nasopharyngeal carcinoma (NPC) is one of the predominant malignancies in South Asian continents, primarily in southern China, Indonesia, and India^1^. According to the global cancer repository report published by GLOBOCAN in 2020 (https://gco.iarc.fr/), NPC ranked 22nd based on occurrence. Among the in-vogue treatment regimens, radiotherapy and parallel chemotherapy increase NPC patients’ survival. To date, identifying NPC at an initial stage is challenging because the symptoms of this cancer resemble other, more common conditions, which creates confusion for clinicians to diagnose. It is also hard to examine the nasopharynx. Sometimes, the submucosal lesions spread silently with a standard appearance during clinical tests. Thus, many NPC patients appear in clinics with advanced stages, resulting in a poor prognosis^2^.

So far, the TNM (tumor–node–metastasis) staging technique is a decisive parameter in providing analytical information and directing treatment choices for cancers, including NPC. To date, this model is not enough to determine the best personalized treatment. About 20–30% of NPC patients with the same stages receiving similar treatment show local relapse or distal metastasis^3–5^, pointing out that molecular subclassification may be clinically appropriate, but more accurate molecular tools are a prerequisite that can stratify patients concerning prognosis and response to therapy. Since NPC is diversely categorized into four subtypes based on histological character and ethnicity-specific occurrence, there is very little knowledge available for NPC’s prognostic biomarkers to predict and improve patient outcomes.

The development of malignancy or its distribution is a complex phenomenon comprising many factors, which may differ uniquely from one cancer to another. Cumulative evidence suggests that the tumors’ molecular characteristics add predictive strength in developing molecular biomarkers. DNA methylation at the 5ʹ cytosine (5mc) was revealed with capabilities to define a better prognosis of various malignancies^6–9^. DNA methylation is one of the most extensive genomic aberrations occurring during carcinogenesis^10^. These abnormalities can broadly be categorized as focal hypermethylation and global hypomethylation^11^. Hypermethylation can promote the silencing the regulatory region of tumor suppressor genes, contributing to cell growth deregulation^12,13^.

On the other side, hypomethylation mainly predominates in the repetitive regions and sometimes unwantedly activates transposable elements within the genome, resulting in further genetic damage^14^. Hypomethylation also drives genomic instability, causing missegregation of chromosomes during cell division^15^. Thus, studying DNA methylation in cancer is a fundamental requirement for comprehending that cancer’s outcome and prognostic behaviour and developing a better treatment regime.

Genome-wide DNA methylation between tissue and whole blood has been investigated in several tumors and displayed a dissimilar portrait of methylation signature, which indicates that blood DNA methylation has an additional role in tumor progression and metastasis over tissue DNA methylation. Moreover, the Study of DNA methylation from blood samples has some advantages over other sampling methods as it is commonly available in epidemiological studies, marginally aggressive, and more suitable to the screening population. Although DNA methylation can be detected in cell-free DNA (cfDNA) from serum or plasma, its low yield and variation in the processing method of isolation indicated significant problems^16^.

However, the underlying relationship between DNA methylation and NPC’s development is poorly understood. Besides two genome-wide DNA methylation studies of primary NPC, only a few biomarkers with diagnostic and prognostic values have been reported^5,17,18^. The only existing report of global blood DNA methylation of head and neck cancer (HNSCC) suggested that environmental risk factors could modify the DNA methylation and are associated with the prognosis of HNSCC. Although this study did not imply the advancement of HNSCC, it led us to hypothesize whether the blood DNA methylation signature differs from tissue DNA methylation in advanced NPC.

Therefore, we aimed to determine the DNA methylation signature from the whole blood of advanced NPC patients and examined the differences in DNA methylation patterns of blood and tissue of NPC patients to identify unique methylation biomarkers. The predictive role of biomarkers in NPC metastasis was investigated further.

## Results

### Blood DNA methyl array data revealed a differential methylation pattern in metastatic NPC

**Figure 1:**
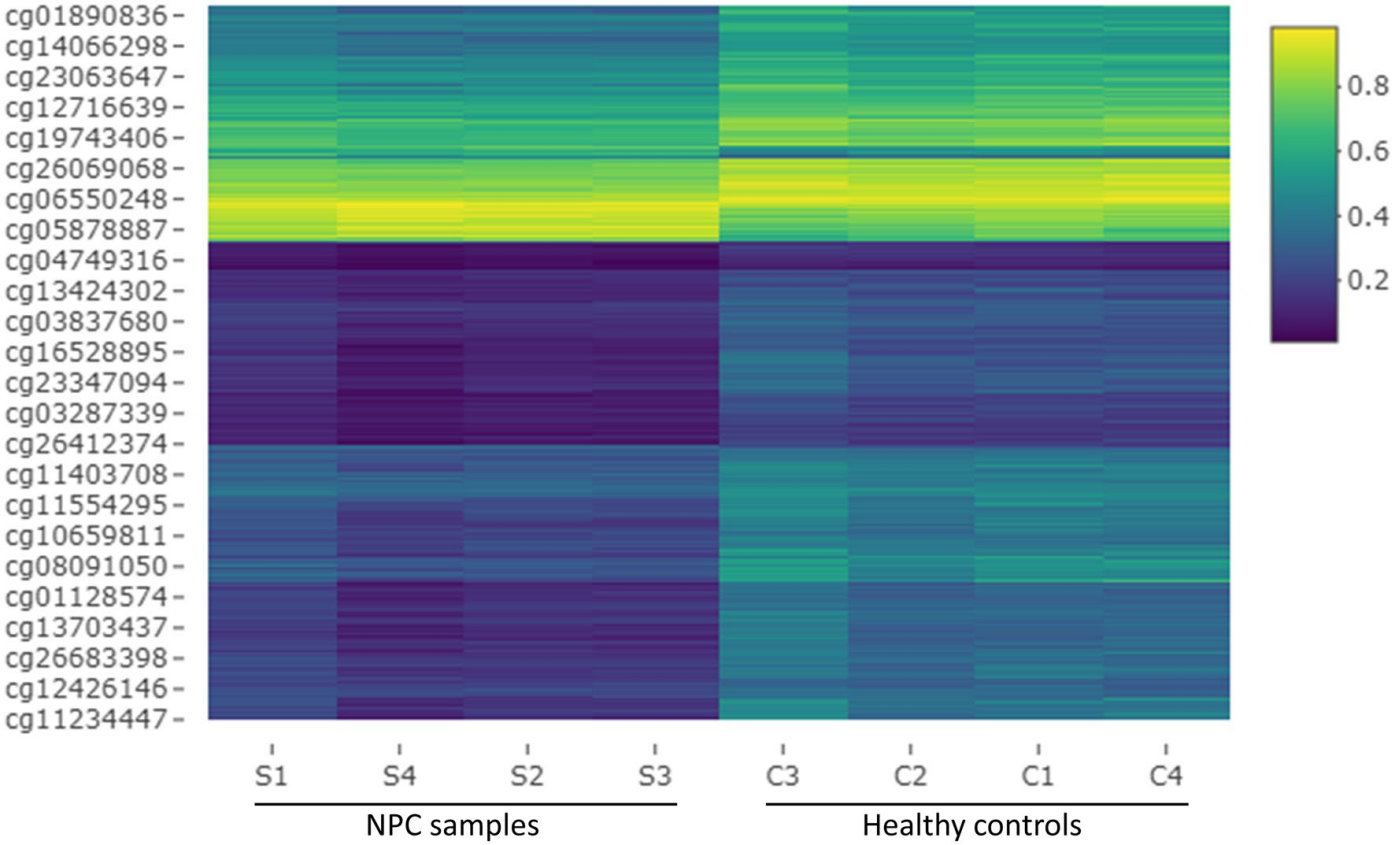
Heatmap of differentially methylated probes (DMPs).

**Figure 2:**
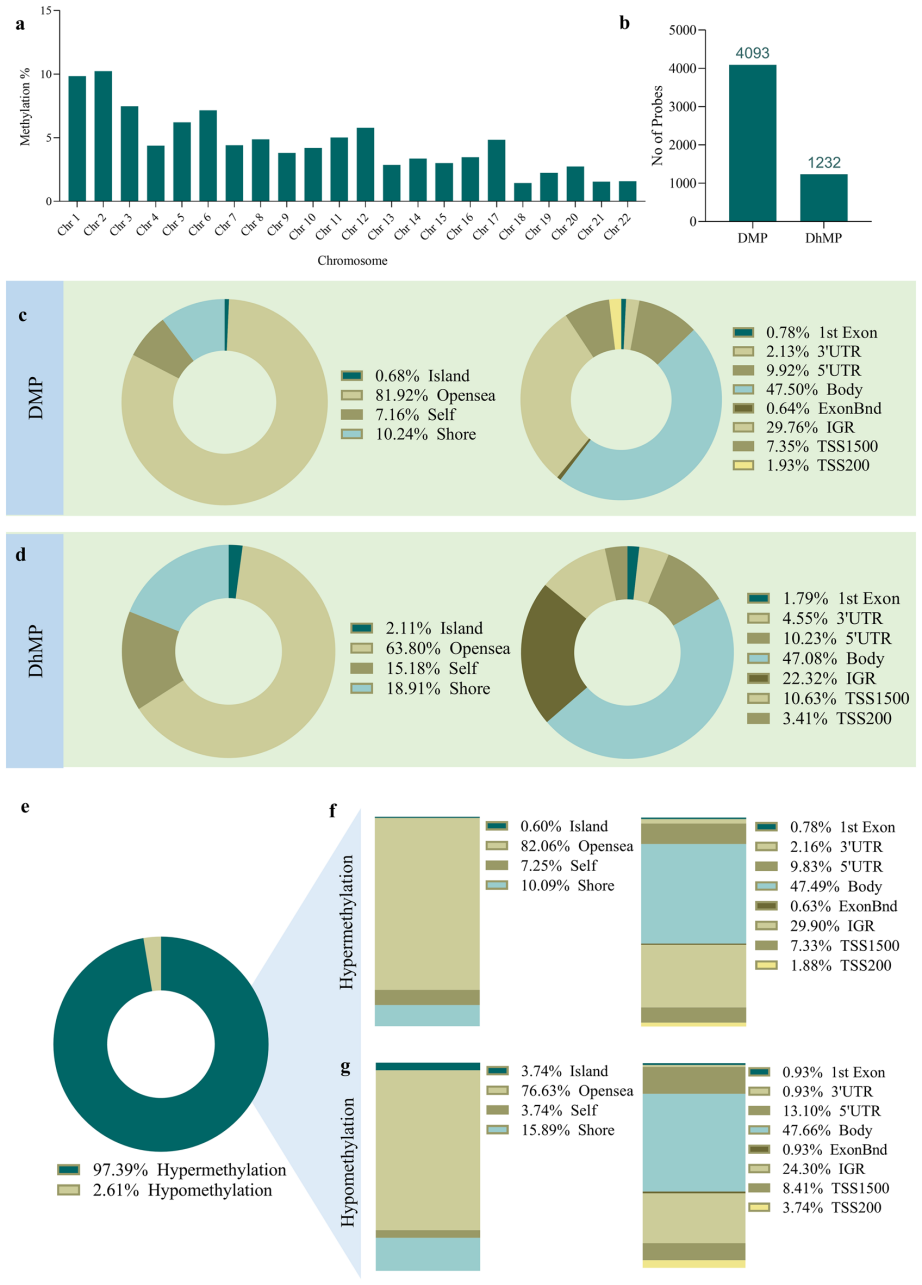
Differential methylation analysis. Genomic regions were annotated as (i) locations related to gene: TSS200 (covers − 200 nucleotide upstream of TSS, generally considered as a proximal promoter); TSS1500 (covers − 200 to − 1500 nucleotide upstream of the transcription start site or TSS, considered as a distal promoter); 5ʹ-UTR and 3ʹUTR (covers the entire untranslated regions); 1st exon; body (Gene Body); ExonBnd (exon boundaries) and IGR (intergenic region). (ii) Locations related to CpG: Island (CpG island, usually extends for 300–3000 base pairs); Shore (up to 2 kb upstream or downstream from CpG island); Shelf (2–4 kb upstream or downstream from CpG island); Open sea (a region that is not associated with CpG island and has not determined yet). (**a**) DMP in different chromosomes; (**b**) the methylation vs hydroxymethylation; (**c**) distribution of DMPs in regions related to genes and CpGs; (**d**) distribution of DhMPs in regions related to genes and CpGs; (**e**) hyper vs hypomethylation; (**f**) distribution of hypermethylation in regions related to genes and CpGs; (**g**) distribution of hypomethylation in regions related to genes and CpGs.

The methylation analysis results are displayed in Figs. 1, 2, Tables S2 and S3. After normalization, probes with Δβ between ≤ 0.2 and ≥ 0.2 were removed, and 4093 probes were identified as DMPs, which covered approximately 2875 genes throughout the genome (Fig. 1 and Table S2). The distribution of DMPs in different chromosomes showed that Chr.1 and 2 were highly methylated (Fig. 2a). Analysis of methylation related to the gene’s location indicated that the highest number of methylations occurred at the gene body (47.50%), followed by the intragenic region or IGR (29.76%) (Fig. 2c left). The open sea region showed comparatively higher DMPs (8.0.92%) than any other locations related to CpG (Fig. 2c right). The majority of the probes were hypermethylated (97.39%) in NPC patients (Fig. 2e). The hypermethylation frequency was highest in the gene body (46.43%), followed by IGR (29.05%). The open sea region was more hypermethylated (80.07%) than other CpG locations.

Methylation in the untranslated region (UTR) is essential in controlling gene expression. In our study, a total of 12.05% hypermethylation was observed in the untranslated region, including 5ʹ-UTR (9.92%) and 3ʹ-UTR (2.13%). Promoter methylation of about 9.28% was noted, in which TSS200 and TSS1500 had 1.93% and 7.35%, respectively (Fig. 2f). The distribution of hypomethylation concerning genes and CpGs followed a similar trend (Fig. 2g).

Unlike 5mc, hydroxymethylation (5hmc) is mainly distributed to the active transcriptional region, accompanied by open chromatin, allowing histone modification. The expression of 5hmc varies from tissue to tissue, and its expression is correspondingly low in cancer stage 4 with metastasis. Patients were shown to live longer with a higher 5hmc level than one with a lower level^19^. Thus, using 5hmc as the ideal prognostic biomarker could be one of the most excellent possibilities for understanding cancer susceptibility. We further conducted the analysis and distribution of 5hmc to understand the ratio of 5hmc to 5mc in advance NPC and found a total of 1232 5hmc probes, which comprised almost one-third of the 5mc (Fig. 2b and Table S3). The highest amount of 5hmc was noticed in the open sea region (82.06%), followed by the shore (18.91%), shelf, and island. The distribution of 5hmc related to gene location exposed the gene body as the highest 5hmc covering region compared to other sites. The UTR contained 14.78% of the 5hmc, in which 5ʹ-UTR and 3ʹ-UTR had 10.23% and 4.55%, respectively (Fig. 2d). A total of 14.01% of 5hmc was noted in the promoter region, including TSS200 (10.63%) and TSS1500 (3.41%).

### Comparative analysis exhibited uniquely methylated probes

**Figure 3:**
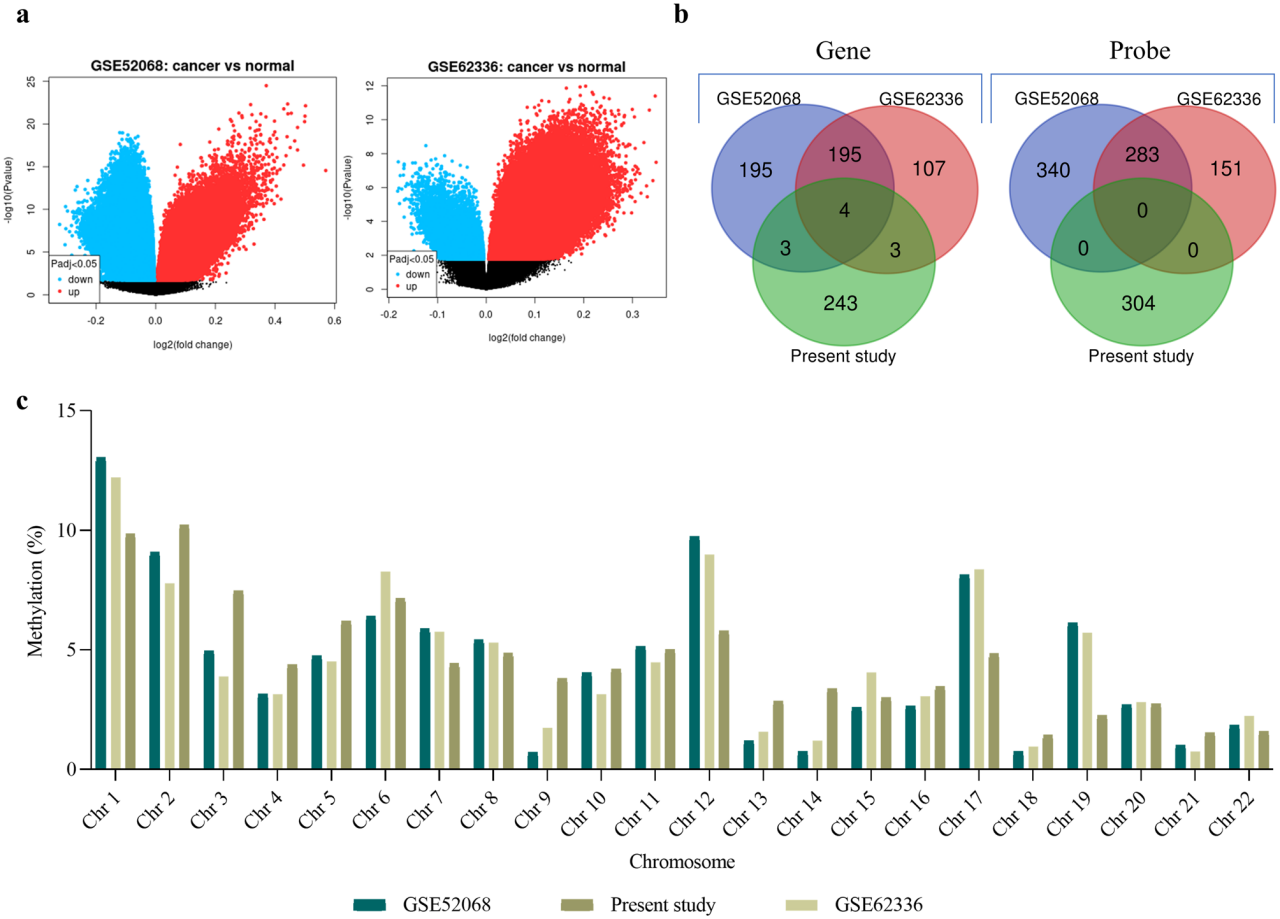
Comparative methylation analysis of the present methyl array data with the existing NPC methyl array GEO dataset. In the first panel, (**a**) shows the volcano plot of two NPC GEO dataset, and (**b**) indicates the comparative analysis through Venn diagram; (**c**) demonstrates the comparative distribution of DMPs in different chromosomes.

The comparative analysis of the present blood DNA methyl array data with the previously existing tissue DNA methylation dataset represented that despite few, most of the genes and probes were uniquely methylated (Fig. 3a,b). The distribution of differential methylation in different chromosomes indicated a discriminative picture. The highest methylation was observed in chromosome 1, above 10%, followed by chromosomes 2 and 19, showing the lowest methylation (< 5%) compared to other datasets (Fig. 3c). The distinct methylation pattern indicated that blood DNA methylation might have an additional role in NPC progression.

Early evidence pointed out that CpG methylation in untranslated regions, promoters, and gene bodies could alter gene expression in many cancers. Recent studies have demonstrated the role of DNA Methylation in the untranslated regions. Methylation in the 5ʹ-UTR regions often upregulates the gene expression. DNA methylation in 3ʹ-UTR is also functionally involved in gene regulation in the pan-cancer network^20–22^. A correlation between CpG methylation in the gene body and cancer prognosis is now established^23^. For further continuation of our study, probes in the CpG island containing hypermethylation and hypomethylation were selected. With this criterion, we found a total of 25 probes that correspond to 22 genes; of these, 21 probes were hypermethylated. The most significant number of hypermethylation and hypomethylation was noted in PLCB3 (Δβ: 0.30, adj. p 0.015) and HLA-DRB5 (Δβ: -0.52, adj. p 0.043) genes, respectively. Among 22 genes, PLCB3, C18orf1, ZNF516, PRKCZ, KDM4B, HLX, MGRN1, UHRF1, SPI1, PLEC1, MPO, FLNB, MLLT1, and HLA-DRB5 resided in the gene body; promoter containing six genes (FGR, COL11A2, SMTN, KCNT1, and APEH); 5ʹ UTR, 3ʹ-UTR and 1^st^ exon were having MBP, ADRBK1 and FUT4 genes respectively. The overall result is depicted in Table 1. The identified probes were cross-validated through TCGA dataset analysis. Data showed that the distribution of DMPs varied according to cancer types, among which cancer BRCA, HNSC, KIRC, KIRP, and UCEC were enriched with statistically significant methylation (p < 0.05).

**Table 1:** Differentially methylated probes in the CpG island in NPC.

Sl. no	CpG	Gene	Δβ	Adj. p value	Chr	Features
1	cg19006947	PLCB3	0.30	0.015	11	Body-island
2	cg04967578	C18orf1	0.20	0.015	18	Body-island
3	cg12019801	ZNF516	0.22	0.015	18	Body-island
4	cg16922167	FGR	0.22	0.017	1	TSS200-island
5	cg18375707	PLCB3	0.28	0.017	11	Body-island
6	cg01891738	FGR	0.21	0.018	1	TSS200-island
7	cg26930596	PRKCZ	0.27	0.018	1	Body-island
8	cg15621731	KDM4B	0.22	0.019	19	Body-island
9	cg09255910	HLX	0.20	0.019	1	Body-island
10	cg00471371	ZNF516	0.29	0.022	18	Body-island
11	cg01662869	MGRN1	0.20	0.024	16	Body-island
12	cg18351781	UHRF1	0.22	0.025	19	Body-island
13	cg15982099	SPI1	0.21	0.026	11	Body-island
14	cg19405177	PLEC1	0.26	0.027	8	Body-island
15	cg02668773	MPO	0.21	0.028	17	Body-island
16	cg08670658	ADRBK1	0.21	0.035	11	3ʹUTR-island
17	cg02266086	COL11A2	0.23	0.035	6	TSS1500-island
18	cg06633438	MLLT1	0.21	0.037	19	Body-island
19	cg10283505	FUT4	0.21	0.037	11	1stExon-island
20	cg25389087	MBP	0.24	0.043	18	5ʹUTR-island
21	cg23730027	FLNB	0.27	0.045	3	Body-island
22	cg01546248	SMTN	− 0.29	0.015	22	TSS1500-island
23	cg22099896	KCNT1	− 0.30	0.016	9	TSS200-island
24	cg01978534	APEH	− 0.22	0.029	3	TSS1500-island
25	cg26981746	HLA-DRB5	− 0.52	0.043	6	Body-island

Adjusted p value ≤ 0.05 is considered as significant.

*Δβ* beta value difference between control and case.

Selected gene characteristics such as coding sequence length, transcript, 5ʹ-UTR, 3ʹ-UTR, genome span, GC content, distribution of gene types, and number of exons were compared with genomes using chi-squared and Student’s t-tests. Data indicated that the identified genes had a statistically significant (p < 0.05) amount of 5ʹ-UTR, an increased percentage of GC content, and chromosome distribution towards the coding region (Fig. S3).

### Functional analysis identified a cancer-specific correlation between DNA methylation and gene expression

**Figure 4:**
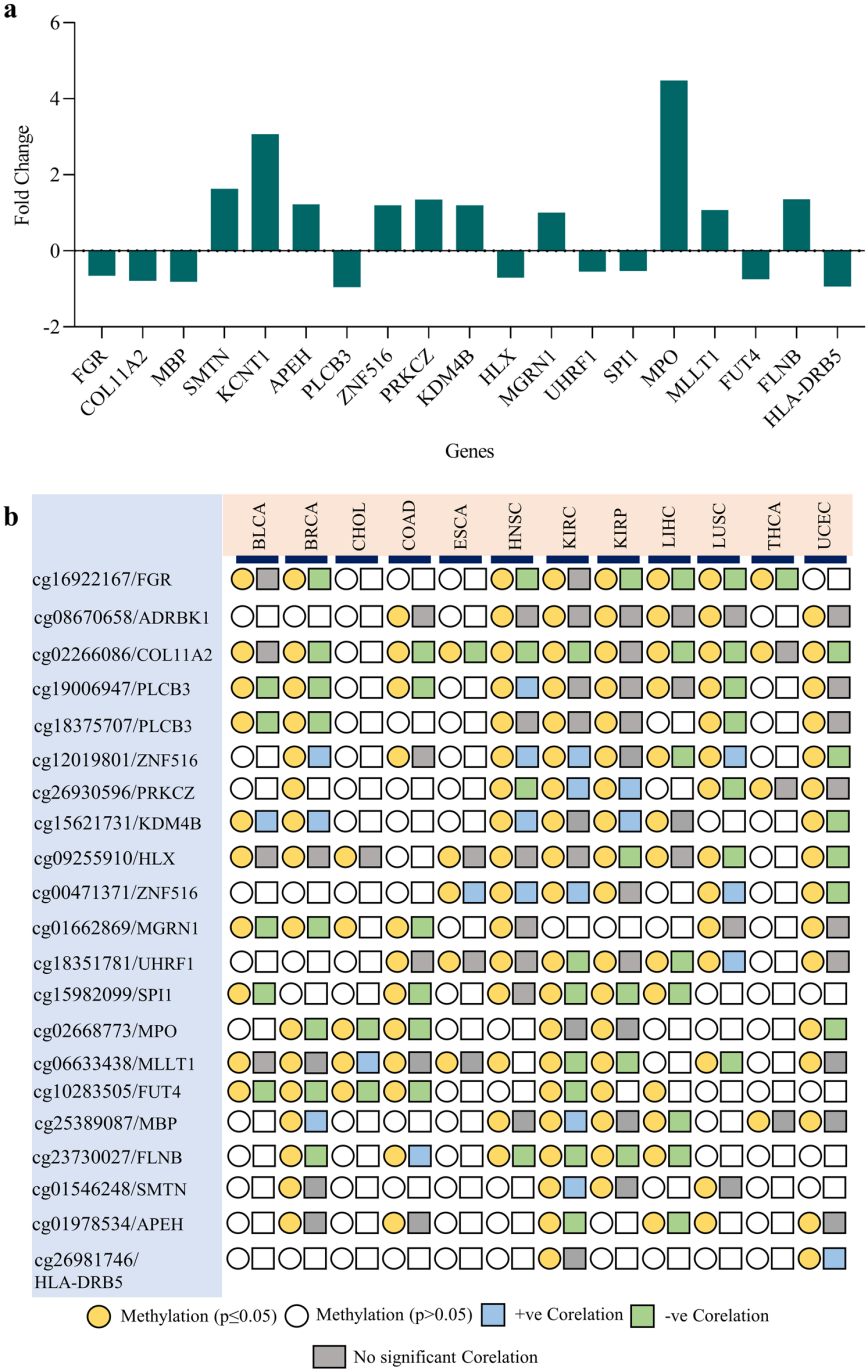
Function analysis of the uniquely methylated probes. (**a**) The RNA-seq. analysis results from GEO dataset and (**b**) shows the result of TCGA methylation analysis and correlation detection between DNA methylation and gene expression of identified probes.

All 22 genes were validated for gene expression by nalysing the RNA sequencing data from the GEO dataset. Fold change was determined by dividing the mean of control FPKM with that of RPKM of cancer. The Data explained that genes SMTN, KCNT1, APEH, ZNF516, PRKCZ, KDM4B, MGRN1, MPO, MLLT1, and FLNB were upregulated. FGR, COL11A2, MBP, PLCB3, HLX, UHRF1, SPI1, FUT4, and HLA-DRB5 were downregulated (Fig. 4a).

We further investigated the correlation between DNA methylation and gene expression by determining the correlation coefficient (r), where r with a positive, negative, and zero value symbolized the positive, negative, and no correlation, respectively. P value ≤ 0.05 was considered significant. Results demonstrated that the methylation of the genes FGR, COLIIA2, APEH, HLX, SPI1, and FUT4 were negatively correlated with gene expression. Similarly, a reverse correlation was observed from the probe of SMTN. These two data were consistent with the RNA-seq result. Conversely, the probe of MGRN1 and MPO did not follow the trend of the RNA-seq data. Additionally, methylation in PLCB3, MBP, ZNF156, PRKCZ, KDM4B, UHRF1, MLLT1, FLNB, and HLA-DRB5 showed both positive and negative associations with gene expression in different tissue-specific carcinomas, suggesting that the methylation and gene expression could be tissue specific. Probes that did not show significant methylation were not included in the correlation analysis. The result of the correlation analysis is depicted in Fig. 4b and Table S4.

### Validation of genome-wide methylation and gene expression data

**Figure 5:**
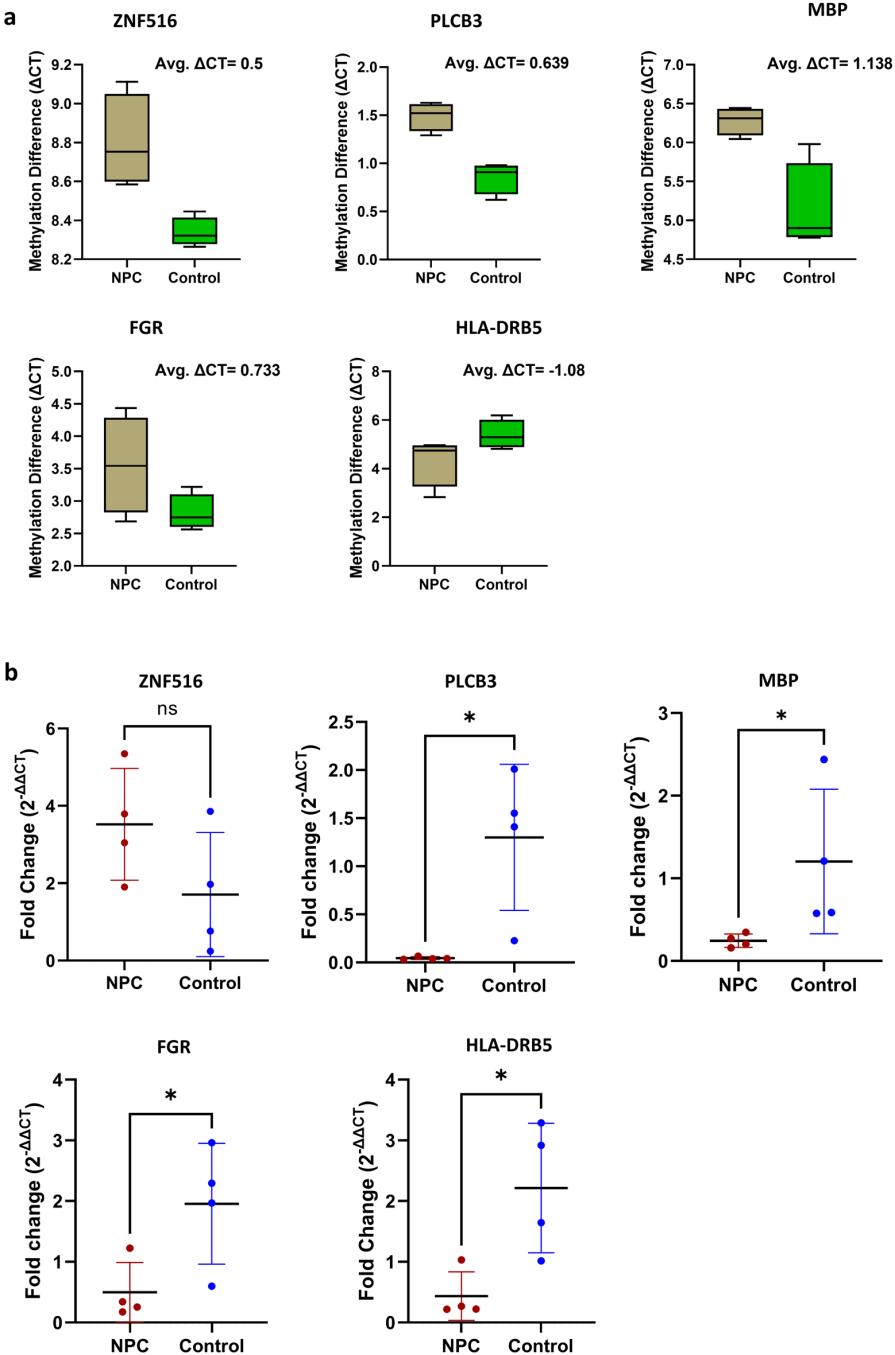
The methyl array and RNA sequence data validation by MSP-RT PCR and quantitative RT-PCR. (**a**) The result of MSP-RT PCR. (**b**) Gene expression by qRT-PCR. P value ≤ 0.05 was considered significant.

To check the accuracy of the methyl array and RNA sequence data, MSP-RT and qRT-PCR for gene expression were employed. MSP has been widely used in clinical settings for the diagnosis and prognosis of diseases such as cancer due to its high specificity, sensitivity, cost, and labor-saving method^24^. Using SYBR-Green technology in real-time PCR enables the objective evaluation of MSP data, providing an easy way to express the methylation status semi-quantitatively without having to perform laborious gel electrophoresis, as shown by existing research^25–27^. Methylation status is quantified by evaluating the difference between the CT values (ΔCT = *u*CT − *m*CT) as described by Yoshioka et.al^28^, where *u* and *m* indicate unmethylation and methylation, respectively. Results of the MSP RT-PCR study revealed that while ZNF516, PLCB3, MBP, and FGR were found to be hypermethylated in NPC (ΔCT_ZNF516_ = 0.5, ΔCT_PLCB3_ = 0.639, ΔCT_MBP_ = 1.138 and ΔCT_FGR_ = 0.733), HLA-DRB5 was hypomethylated. The result of the gene-wise methylation difference and melting curve of each gene is depicted in Figs. 5a and S5, respectively. Gene expression study showed a significantly lower expression of RNA in PLCB3, MBP, and FGR, HLA-DRB5 (p ≤ 0.05) and a higher expression in ZNF516 (Fig. 5b).

### DNA Methylation in CpG harbors several transcription factor binding sites

**Figure 6:**
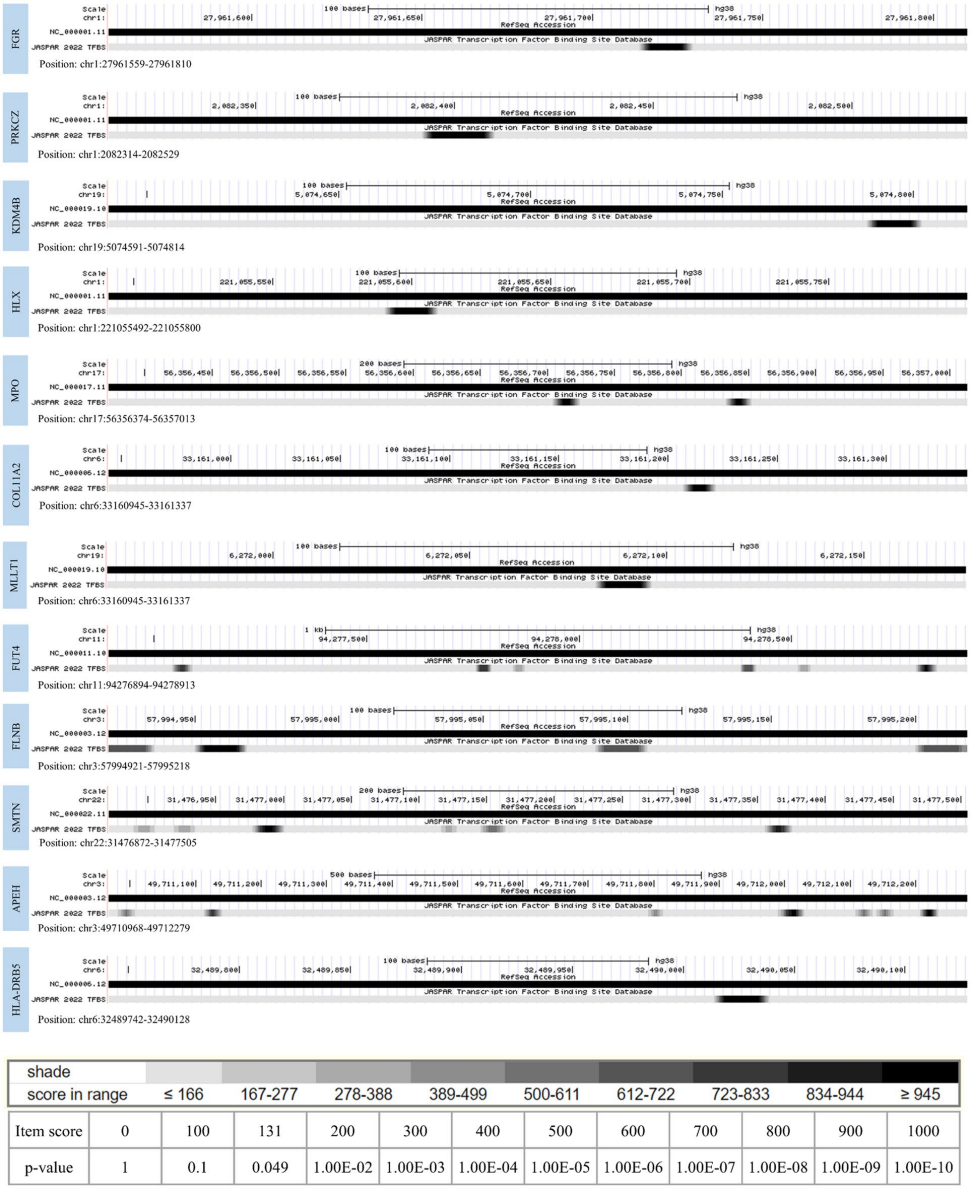
Prediction for transcription factor binding site in the region of methylation.

Transcription factor takes the overall control of gene regulation, thus maintaining normal cellular functions. They are commonly dysregulated in many human cancers and are associated with approximately 20% of oncogenesis^29^. DNA methylation, mainly in the CpG region, potentially alters TFs binding to DNA and promotes gene regulation modification^30^. We predicted TFBSs present in the CpG island of designated probes. TFs were selected according to the PWM relative score ≥ 0.8 and a p-value < 0.05. The shading of the boxes indicated the p-value of the profile’s match to that position (scaled between 0 and 1000 scores, where 0 corresponds to a p-value of 1 and 1000 to a p-value ≤ 10–10); thus, the darker the shade, the lower (better) the p-value. The predicted score was adjusted to 600, and TFs with a score more than 600 were obtained. Data indicated that the identified genomic region of FUT4 and APEH were enriched with several TF binding sites that scored ranging from 963 to 611. Moreover, binding sites for Zinc finger proteins (ZBTB18, ZNF680, ZNF460, ZNF135, ZNF384, ZNF354A, Zic3, Zic1) and FOX family TFs (FOXD3 and FOXO1) were mostly observed. TFs in the ZNFs and FOX family modulates major regulatory pathways and involve in the onset of cancers. The result of TFBS prediction is depicted in Fig. 6 and Table S7.

### Methylation in CpG could modulate immunological functions in various cellular pathways

**Figure 7:**
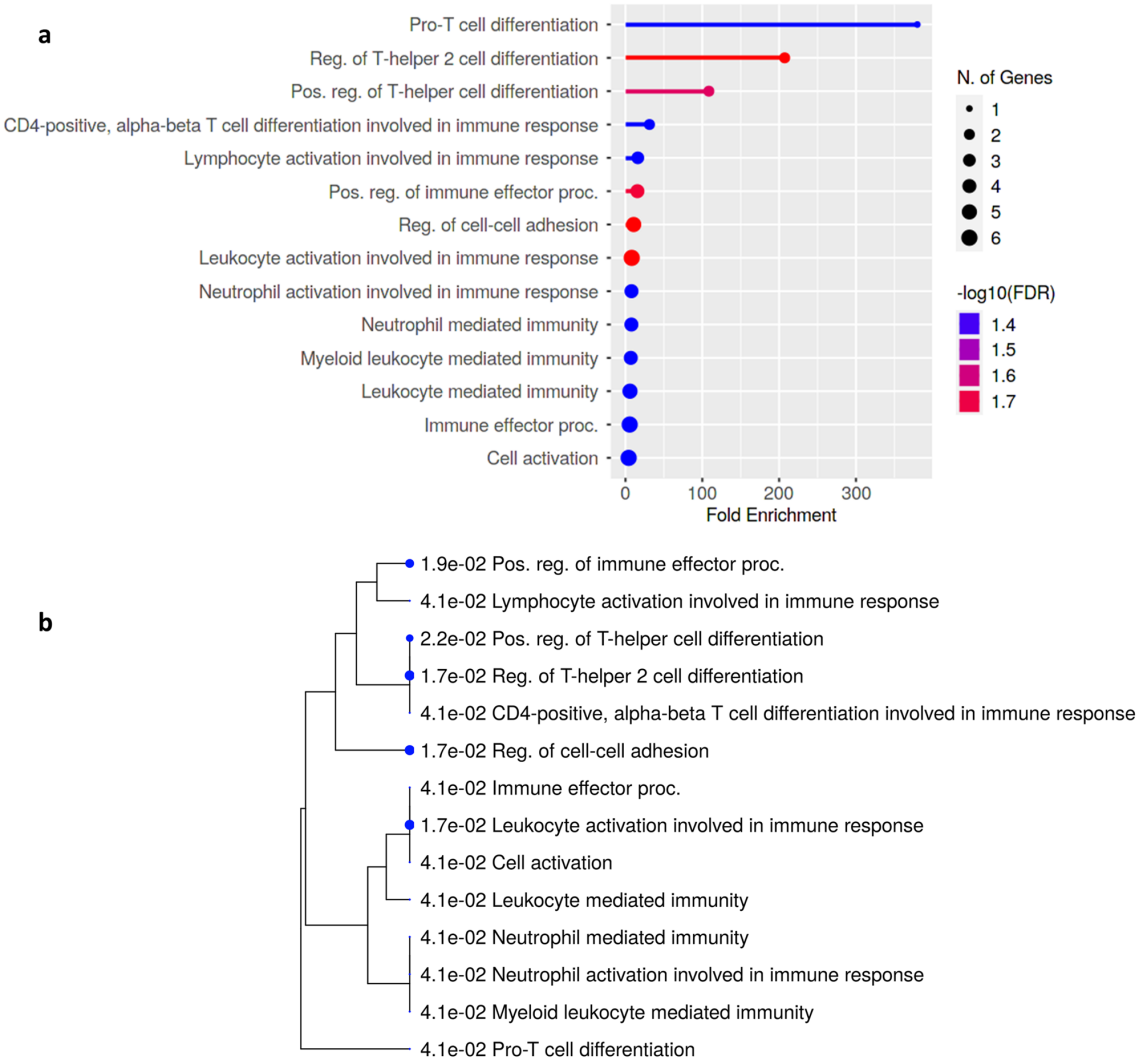
Gene enrichment analysis. (**a**) The enrichment plot, and (**b**) indicates the hierarchical clustering tree summarizes the correlation among significant pathways.

**Figure 8:**
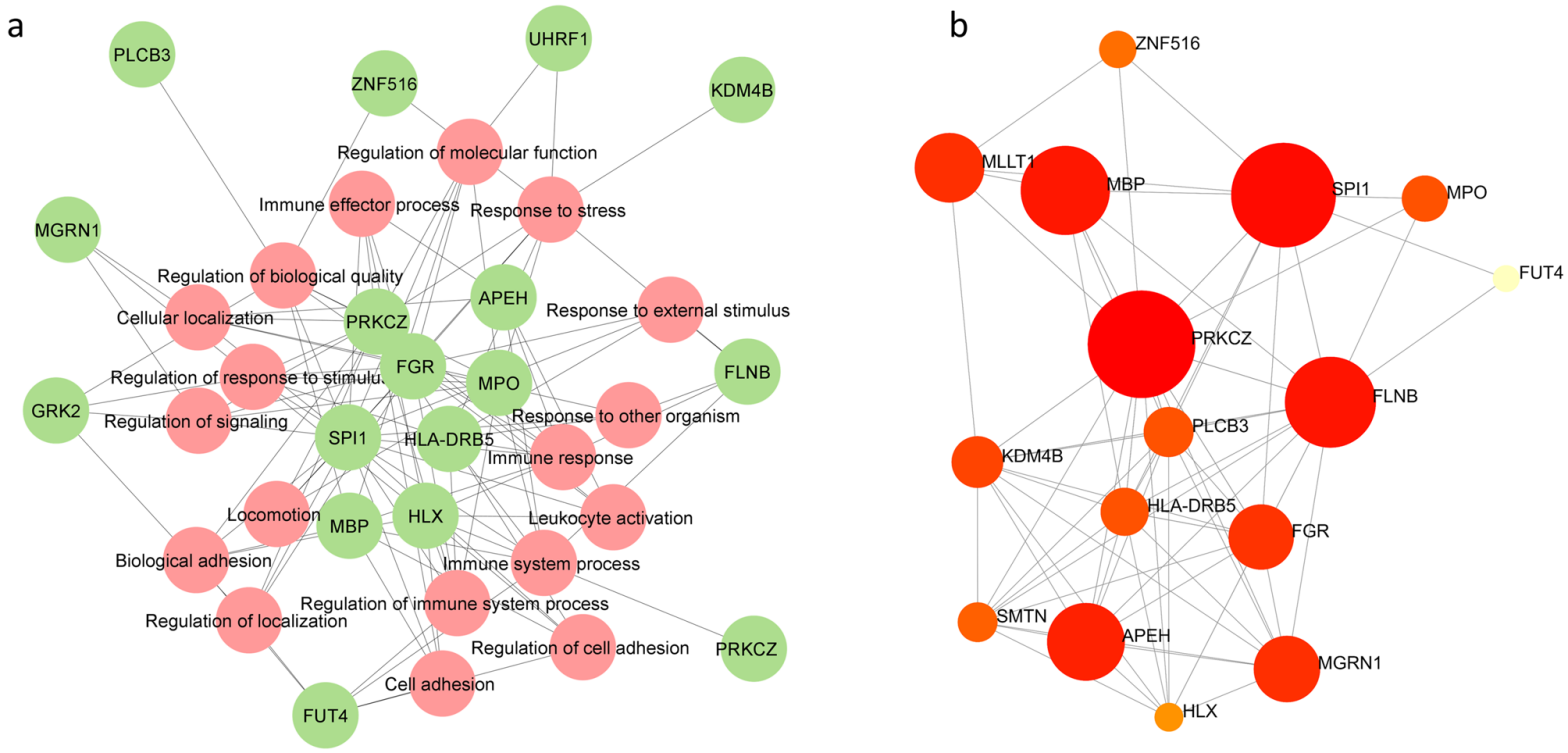
Network analysis: (**a**) indicates the interaction of identified genes with biological pathways. (**b**) Describes the interconnection among the genes.

Pathway enrichment study is a statistical method of exploring pathways enriched in the input gene list relative to what is expected by chance^31^. The enrichment analysis results are displayed in Fig. 7 and Table S8. After the analysis, pathways were filtered based on a user-specified FDR cut-off, followed by shorting the significant pathway by FDR, Fold Enrichment, or other metrics. Results indicated that genes were connected with different immune-modulatory functions, such as T-cell differentiation, cell–cell adhesion, neutrophil, and leucocyte-mediated immunity etc. (Fig. 7a). A hierarchical clustering tree was created to comprehend the correlation among significant pathways listed in the enrichment tab, in which bigger dots indicate more significant P-values. Data showed that T-cell differentiation, cell–cell adhesion, and leucocyte activation were potentially augmented (Fig. 7b). We further generated the interactive plot to establish the intricate relationship between genes and enriched pathways (Fig. 8a). The Gene interaction network was constructed to check the protein–protein interaction among the selected genes; a bigger node indicates a significant interaction (Fig. 8b).

## Discussion

So far, the study of DNA methylation in NPC has mainly focused on tumor suppressor genes’ methylation patterns^17^. Existing genome-wide DNA methylation reports deciphered high methylation among a Chinese population. The report suggested that this could affect the current therapy in NPC patients^5^. Another methylome study explained increased methylation at chromosome 6p in the same ethnic population^18^. Recently, peripheral blood methylation signatures in various solid tumors exhibited a distinct epigenetic signature, indicating cancer’s unique prognostic and diagnostic biomarkers and their resistance to therapy^16,32–34^. Blood DNA methylation in head and neck cancer (HNSCC) was first reported in 2006. It explored an independent association between hypomethylation and HNSCC prognosis and displayed a complex relationship with the known risk factors associated with this cancer^35^. This growing evidence indicated the additional role of blood DNA methylation on cancer progression, metastasis, and recurrence.

Our current study exhibited a different pattern of methylation in NPC. Chromosomes 1 and 2 have the highest methylation, indicating a distinctive signature from NPC tissue DNA methylation. According to previous reports, the gene body’s methylation represses intragenic transcription and allows efficient transcriptional elongation^23,36^. Our data suggested that the distribution of DMPs in the location relative to the gene and CpG showed increased methylation in the gene body and the open sea region. A similar pattern was also found in other cancers. A higher amount of hypermethylation in the NPC patients’ samples was observed in this study. This finding was consistent with the previous reports published in China, signifying that hypermethylation is comparatively higher in NPC than in other cancers^18^.

CpG methylation is a primary concern of cancer risk. The Discovery of hypermethylation in the CpG island of certain tumor suppressor genes interrupts many cellular pathways like DNA repair, apoptosis, cell cycle control, cell–cell adhesion, etc.^37^. Unlike hypermethylation, hypomethylation in CpG comes later in cancers and contributes to metastatic tumor heterogeneity^38^. We found twenty-two unique genes (corresponding to twenty-five probes) that were differentially methylated in their CpG islands. The functional study analyzing RNA sequence revealed the differential expression of those genes in NPC, and TCGA analysis further reinforced the study. The results of the genome-wise methyl array and RNA sequence were further cross-validated by performing MSP-RT PCR and quantitative RT-PCR for gene expression; both exhibited consistency with the previous data, suggesting the accuracy and robustness of the methylation analysis.

Moreover, a correlation was detected between methylation of the specific probe and gene expression in cancers other than NPC. This study also implied that the methylation within the particular region of CpG and gene expression could vary according to cancer. Further, the analysis of TFBS of CpG methylated promoter and gene bodies illustrated binding sites for several essential TFs such as ZNFs and FOXs. Early reports demonstrated that ZNF and FOX family TFs are crucial in cell migration and proliferation^39,40^. A differential expression of other TFs investigated in many cancers is associated with tumors’ development, spread, invasiveness, and lack of proper immune responses^41–45^. It can be proposed that CpG methylation could alter the binding of TFs, which is thought to modify the gene expression of the corresponding proteins, resulting in NPC advancement. However, further research is needed to imply the hypothesis.

Additionally, the gene enrichment analysis of the identified genes was connected to various cellular pathways and immune activations. Previous research projected the contribution of these genes in different cancers like breast, pancreatic, and ovarian. Genes PRKCZ (Protein Kinase C Zeta), FGR (Src family protein), KDM4B, MPO, COL11A1, FUT4 (Fucosyltransferase IV), and APEH (acylpeptide hydrolase) are directly associated with tumor aggressiveness, growth, and migration, and invasiveness, resulting in poor clinical outcome in cancer patients^41,46–50^. Similarly, HLX (H2.0-like homeobox) controls early hematopoiesis and promotes acute myeloid leukemia^51^, and HLA-DRB5 (HLA major histocompatibility complex, class II, DR beta 5) acts as a receptor for T cell activation. Its expression is shown to be upregulated in breast cancer^52^.

The present study elucidated a lucid picture of genome-wide blood DNA methylation of metastatic NPC, which could set a goal and a future direction of developing more precise molecular tools based on methylation biomarkers for detecting and diagnosing primary and advanced NPC. Though the study was statistically robust and comprehensive, a few limitations exist. Limited sample size could affect statistical interpretation, and a lack of downstream experiments might create a lag for further validation.

## Methods

### Study selection

Blood samples from four NPC patients were collected from the Eden Medical Centre, Dimapur, Nagaland, India, between 2018 and 2019. For the clinical examination, detailed medical history, physical check-up, Nasopharyngioscopy, serum biochemistry, blood count, chest X-ray, CT or MRI examination of the nasopharynges, skull base, and any suspect metastatic sites (paranasal sinuses) were tested. All the patients suffered from non-keratinizing undifferentiated carcinoma (NKUC). NPC types were determined following the guidelines of WHO^53^. TNM classification was confirmed according to the recommendation of the American Joint Committee on Cancer (AJCC)^54,55^. All the samples were obtained before the start of the diagnosis. Four control samples were obtained separately from matched disease-free healthy individuals’ age, sex, and ethnicity. Both cases and controls tested negative for Epstein Bar Virus (EBV). Written informed consent was obtained from all the patients. Ethical approval was obtained from the ethics committee of Visva-Bharati University, West Bengal and Eden Medical centre, Dimapur, Nagaland, as the participating institute. All methods were performed in accordance with the relevant guidelines and regulations. Demographic and Clinical characteristics of NPC cases and controls are shown in Table S1.

### Methylation profiling and data processing

Genomic DNA was extracted from the whole blood of eight samples, followed by the bisulfite conversion using the EZ DNA methylation Gold Kit (Zymo Research, USA). The whole-genome methylation array was carried out using the Infinium Methylation EPIC BeadChip Kit (Illumina Inc, USA). The array data (IDAT files) were nalysed using the ChAMP Bioconductor package in R studio (Version 1.2.5042)^56^. Based on the methylation intensities, the methylation level was measured by the beta-value (β) method, ranging from 1 to 0 (where the value of 1 indicates a fully methylated probe and 0 indicates an unmethylated probe)^57^. Β value is calculated as the ratio of the methylated probe intensity to the overall intensity (sum of methylated and unmethylated probe intensities)^11^.

**Figure 9:**
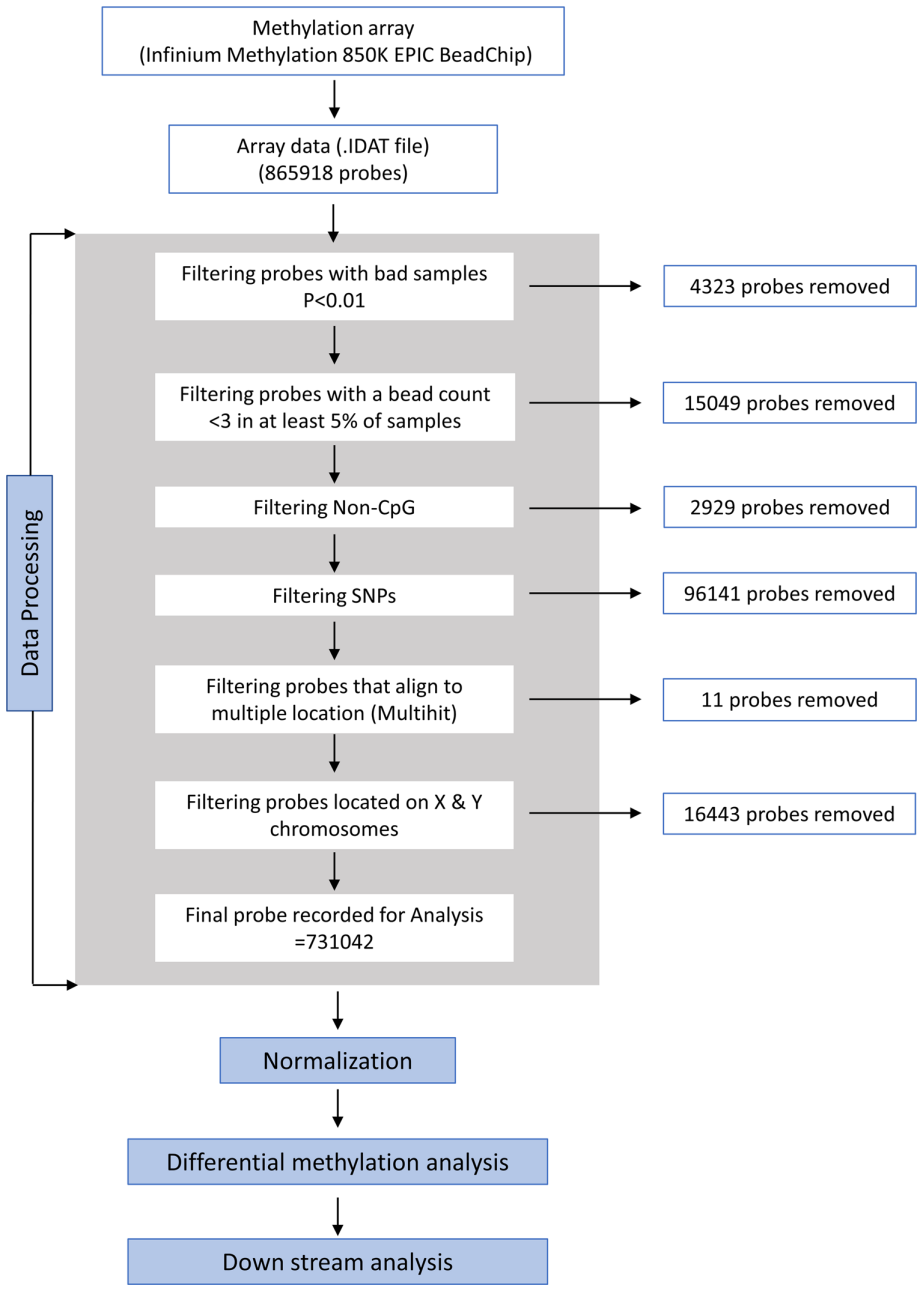
Schematic diagram of methylation data processing.

From the methylation EPIC BeadChip, 865,918 methylated probes were detected initially. Probes having bad samples (p > 0.01), a bead count of < 3, having non-CpGs, containing SNPs, probes aligned to multiple locations, and those located at X and Y chromosomes were removed. Eventually, 731,042 methylated probes were recorded. The result of the quality control (QC), beta value distribution plot, and Frequency polygon is shown in Fig. S1. The beta-mixture quantile normalization (BMIQ) method was used to adjust the probe type or color bias, subtract background signals, and eliminate systematic errors (Fig. S2). It is a well-established normalization method that decomposes the β profiles of Type I and Type II probes into two mixtures of 3 methylation states. Then quantile normalized the three distributions of Type II profile corresponding to those of Type I profile^58^. Wilcoxon signed-rank test was performed for each locus of the sample group (Cancer vs. Control) to identify a significantly differential methylated locus. Multiple testing correction was performed over p values using the Benjamini-Hochberg (BH) method. The cut-off for differential methylation was restricted to BH-adjusted p-values < 0.05 and absolute beta-value (mean beta-value difference between cancer and control) or Δβ ≥ 0.2. CpG was considered hypermethylated or hypomethylated if the Δβ was ≥ 0.2 or ≤ 0.2, respectively^59,60^. The outline of the overall process is depicted in Fig. 9.

### Comparative analysis of methyl array data with global NPC methylation dataset

The present methyl array data were compared with available global NPC methyl array data (GSE52068 and GSE62336) deposited in the NCBI-GEO database (https://www.ncbi.nlm.nih.gov/gds). Data from GEO datasets were retrieved and re-analyzed by ‘GEOquery’ and ‘Limma’ R Bioconductor packages. Genes and CpG probes from all the datasets were compared separately by a Venn diagram to view common and unique genes or probes.

### RNA-Seq and TCGA data analysis

RNA-sequencing data of two NPC and one para-cancerous tissue was retrieved from the GEO database (GSE134886) and re-analyzed to inspect the gene expression. Comprehensive methylation analysis was performed between the differentially methylated probes (DMPs) of NPC and other carcinomas existed in the Cancer Genome Atlas Program (TCGA): bladder urothelial carcinoma (BLCA, n = 424), breast invasive carcinoma (BRCA, n = 853), cholangiocarcinoma (CHOL, n = 45), colon adenocarcinoma (COAD, n = 288), esophageal carcinoma (ESCA, n = 190), head and neck squamous cell carcinoma (HNSC, n = 535), kidney renal clear cell carcinoma (KIRC, n = 333), kidney renal papillary cell carcinoma (KIRP, n = 292), liver hepatocellular carcinoma (LIHC, n = 409), lung squamous cell carcinoma (LUSC, n = 370), thyroid carcinoma (THCA, n = 558), and uterine corpus endometrial carcinoma (UCEC, n = 187). Further, the Pearson method was set to calculate the corresponding correlation coefficient to conduct a pair-wise correlation analysis between methylation and gene expression.

### Bisulfite conversion, methylation-specific real-time PCR and quantitative RT-PCR

Bisulfite conversion was performed by EpiJET Bisulfite Conversion Kit (Thermo Scientific, USA) following the manufacturer’s instruction. During the conversion process, methylated cytosine remains unchanged, while unmethylated cytosine is converted into uracil. Two types of primers and the SYBR Green qPCR Master Mix were utilized for MSP-RT. CpG island was identified for each gene of interest (Fig. S4), followed by designing primers specifically for the site of the methylation-favoured regions of individual genes. One primer set was designed for fully methylated sequences that recognize unconverted cytosine during bisulfite treatment. In contrast, other primer sets recognize fully unmethylated sequences and bind to uracil instead of cytosine (Table S5). RT-PCR was performed under the following conditions: An initial denaturation at 95 °C for 5 min, then 35 cycles of 95 °C for 30 s, 50–55 °C (varied for different genes primer) for 30 s, 72 °C for 1 min, and a final step at 72° for 5 min.

RNA was extracted from NPC and control samples using QIAamp RNA Blood Mini Kit (Qiagen, USA) following the manufacturer’s protocol to validate the RNA sequence data for gene expression. After cDNA synthesis, RT-PCR was carried out at 95 °C for 3 min, followed by 40 cycles of denaturation at 95 °C for 30 s, annealing at 55–60 °C (varied for different genes primer) for 30 s, and amplification at 72 °C for 1 min. Comparing CT values of NPC and control, fold change was calculated. Primers for the qRT-PCR are listed in Table S6.

### Transcription factor binding site prediction

For the prediction of transcription factor binding sites (TFBS) of the methylation-specific probes, the JASPAR 2022 module of the UCSC Genome Browser was employed^61^. JASPAR CORE TF-binding profiles read each taxon independently using position weight matrix (PWM) Scanning and generate a score for each TF.

### Gene ontology and interaction network analysis

Gene ontology analysis (GO) was performed with an FDR cut-off < 0.05 using the open-source server ShinyGO 0.76^62^. It accesses the Ensembl and pathway database from many other sources and uses many R packages to visualize and analyze a gene’s relative functions. FDR was calculated based on the nominal P-value from the hypergeometric test. Depending on the FDR-specified cut-off, Pathways were filtered. Significant pathways were sorted by FDR, Fold Enrichment, or other metrics. The preferred GO ontology functions were selected, viz., biological process, molecular function, and cellular component.

Using the integrated CHAMP-FEM package, we identified the connected subnetwork of the protein interaction of a differentially methylated promoter region with a large average edge-weight density. Weight edges were constructed from the statistical association of DNA methylation with the phenotype of interest (case vs. control), where uniquely methylated genes were considered as seeds for the network construction. Queries were uploaded onto NetworkAnalyst (version 3.0), a comprehensive statistical network visual analytics platform^63^. IMEx Interactome database was selected, which uses literature-curated complete data from InnateDB^64^.

### Ethics declarations

Obtained from Visva-Bharati and the participating institute.

### Consent to participate

Informed consent was taken from each patient.

### Supplementary Information

Supplementary Information.

## Data Availability

The data generated in this study are available within the article and its supplementary data files S1. Expression profile data analysed in this study were obtained from Gene Expression Omnibus (GEO) at GSE52068, GSE62336 and GSE134886.

## References

[1] Mahdavifar N, Ghoncheh M, Mohammadian-Hafshejani A, Khosravi B, Salehiniya H (2016). Epidemiology and inequality in the incidence and mortality of nasopharynx cancer in Asia. Osong. Public Health Res. Perspect.

[2] Abdullah B, Alias A, Hassan S (2009). Challenges in the management of nasopharyngeal carcinoma: A review. Malays J. Med. Sci..

[3] Chua DTT (2005). Long-term survival after cisplatin-based induction chemotherapy and radiotherapy for nasopharyngeal carcinoma: A pooled data analysis of two phase III trials. J. Clin. Oncol..

[4] Chen L (2012). Concurrent chemoradiotherapy plus adjuvant chemotherapy versus concurrent chemoradiotherapy alone in patients with locoregionally advanced nasopharyngeal carcinoma: A phase 3 multicentre randomised controlled trial. Lancet Oncol..

[5] Jiang W (2015). Genome-wide identification of a methylation gene panel as a prognostic biomarker in nasopharyngeal carcinoma. Mol. Cancer Ther..

[6] Brock MV (2008). DNA methylation markers and early recurrence in stage I lung cancer. N. Engl. J. Med..

[7] de Maat MF (2008). Quantitative analysis of methylation of genomic loci in early-stage rectal cancer predicts distant recurrence. J. Clin. Oncol..

[8] Kuang Y (2020). Genome-wide analysis of methylation-Driven genes and identification of an eight-gene panel for prognosis prediction in breast cancer. Front. Genet..

[9] Mori T (2005). Predictive utility of circulating methylated DNA in serum of melanoma patients receiving biochemotherapy. J. Clin. Oncol..

[10] Locke WJ (2019). DNA methylation cancer biomarkers: Translation to the clinic. Front Genet.

[11] Ross JP, Rand KN, Molloy PL (2010). Hypomethylation of repeated DNA sequences in cancer. Epigenomics.

[12] Ehrlich M (2002). DNA methylation in cancer: too much, but also too little. Oncogene.

[13] Stone A (2015). DNA methylation of oestrogen-regulated enhancers defines endocrine sensitivity in breast cancer. Nat. Commun..

[14] Daskalos A (2009). Hypomethylation of retrotransposable elements correlates with genomic instability in non-small cell lung cancer. Int. J. Cancer.

[15] Prada D (2012). Satellite 2 demethylation induced by 5-azacytidine is associated with missegregation of chromosomes 1 and 16 in human somatic cells. Mutat. Res..

[16] Raut, J. R., Guan, Z., Schrotz-King, P. & Brenner, H. Whole-blood DNA methylation markers for risk stratification in colorectal cancer screening: A systematic review. *Cancers (Basel)* **11**. 10.3390/cancers11070912 (2019). 10.3390/cancers11070912 PMC6678372 31261771

[17] Jiang W, Cai R, Chen QQ (2015). DNA methylation biomarkers for nasopharyngeal carcinoma: Diagnostic and prognostic tools. Asian Pac. J. Cancer Prev..

[18] Dai W (2015). Comparative methylome analysis in solid tumors reveals aberrant methylation at chromosome 6p in nasopharyngeal carcinoma. Cancer Med..

[19] Xu T, Gao H (2020). Hydroxymethylation and tumors: Can 5-hydroxymethylation be used as a marker for tumor diagnosis and treatment?. Hum Genom..

[20] McGuire MH (2019). Pan-cancer genomic analysis links 3’UTR DNA methylation with increased gene expression in T cells. EBioMedicine.

[21] Rauluseviciute I, Drabløs F, Rye MB (2020). DNA hypermethylation associated with upregulated gene expression in prostate cancer demonstrates the diversity of epigenetic regulation. BMC Med. Genom..

[22] Evans DGR (2018). A dominantly inherited 5' UTR variant causing methylation-associated silencing of BRCA1 as a cause of breast and ovarian cancer. Am. J. Hum. Genet..

[23] Arechederra M (2018). Hypermethylation of gene body CpG islands predicts high dosage of functional oncogenes in liver cancer. Nat. Commun..

[24] Nassar FJ, Msheik ZS, Nasr RR, Temraz SN (2021). Methylated circulating tumor DNA as a biomarker for colorectal cancer diagnosis, prognosis, and prediction. Clin. Epigenet..

[25] Vlassenbroeck I (2008). Validation of real-time methylation-specific PCR to determine O6-methylguanine-DNA methyltransferase gene promoter methylation in glioma. J. Mol. Diagn..

[26] Ogino S (2006). Precision and performance characteristics of bisulfite conversion and real-time PCR (MethyLight) for quantitative DNA methylation analysis. J. Mol. Diagn..

[27] Hattermann K, Mehdorn HM, Mentlein R, Schultka S, Held-Feindt J (2008). A methylation-specific and SYBR-green-based quantitative polymerase chain reaction technique for O6-methylguanine DNA methyltransferase promoter methylation analysis. Anal. Biochem..

[28] Yoshioka M (2018). Real-time methylation-specific PCR for the evaluation of methylation status of MGMT gene in glioblastoma. Oncotarget.

[29] Vishnoi, K., Viswakarma, N., Rana, A. & Rana, B. Transcription factors in cancer development and therapy. *Cancers (Basel)* **12**, 1. 10.3390/cancers12082296 (2020). 10.3390/cancers12082296 PMC7464564 32824207

[30] Héberlé É, Bardet AF (2019). Sensitivity of transcription factors to DNA methylation. Essays Biochem..

[31] Reimand J (2019). Pathway enrichment analysis and visualization of omics data using g:Profiler, GSEA. Cytosc. Enrichment Map. Nat. Protoc.

[32] Xu Z, Sandler DP, Taylor JA (2020). Blood DNA methylation and breast cancer: A prospective case-cohort analysis in the sister study. J. Natl. Cancer Inst..

[33] Kao WY (2017). Genome-wide identification of blood DNA methylation patterns associated with early-onset hepatocellular carcinoma development in hepatitis B carriers. Mol. Carcinog..

[34] Dong L, Ren H (2018). Blood-based DNA methylation biomarkers for early detection of colorectal cancer. J. Proteom. Bioinform..

[35] Hsiung DT (2007). Global DNA methylation level in whole blood as a biomarker in head and neck squamous cell carcinoma. Cancer Epidemiol. Biomarkers Prev..

[36] Jjingo D, Conley AB, Yi SV, Lunyak VV, Jordan IK (2012). On the presence and role of human gene-body DNA methylation. Oncotarget.

[37] Esteller M (2002). CpG island hypermethylation and tumor suppressor genes: A booming present, a brighter future. Oncogene.

[38] Yegnasubramanian S (2008). DNA hypomethylation arises later in prostate cancer progression than CpG island hypermethylation and contributes to metastatic tumor heterogeneity. Cancer Res..

[39] Jen J, Wang YC (2016). Zinc finger proteins in cancer progression. J. Biomed. Sci..

[40] Katoh M, Igarashi M, Fukuda H, Nakagama H, Katoh M (2013). Cancer genetics and genomics of human FOX family genes. Cancer Lett..

[41] Seto KKY, Andrulis IL (2015). Atypical protein kinase C zeta: Potential player in cell survival and cell migration of ovarian cancer. PLOS ONE.

[42] Xiao, Z. J. *et al.* NFATc2 enhances tumor-initiating phenotypes through the NFATc2/SOX2/ALDH axis in lung adenocarcinoma. *Elife* **6**. 10.7554/eLife.26733 (2017). 10.7554/eLife.26733 PMC5570574 28737489

[43] Tian M (2020). IRF3 prevents colorectal tumorigenesis via inhibiting the nuclear translocation of β-catenin. Nat. Commun..

[44] Lee CJ (2020). Stat2 stability regulation: An intersection between immunity and carcinogenesis. Exp. Mol. Med..

[45] Li F, Wang T, Tang S (2015). SOX14 promotes proliferation and invasion of cervical cancer cells through Wnt/β-catenin pathway. Int. J. Clin. Exp. Pathol..

[46] Kim HS (2011). Functional roles of Src and Fgr in ovarian carcinoma. Clin. Cancer Res..

[47] Wilson, C. & Krieg, A. J. KDM4B: A nail for every hammer? *Genes (Basel)* **10**. 10.3390/genes10020134 (2019). 10.3390/genes10020134 PMC6410163 30759871

[48] Nallanthighal, S., Heiserman, J. P. & Cheon, D. J. Collagen type XI alpha 1 (COL11A1): A novel biomarker and a key player in cancer. *Cancers (Basel)* **13**. 10.3390/cancers13050935 (2021). 10.3390/cancers13050935 PMC7956367 33668097

[49] Yan, X., Lin, Y., Liu, S., aziz, F. & Yan, Q. Fucosyltransferase IV (FUT4) as an effective biomarker for the diagnosis of breast cancer. *Biomed. Pharmacother.* **70**, 299–304. 10.1016/j.biopha.2014.12.048 (2015). 10.1016/j.biopha.2014.12.048 25776515

[50] Scaloni A (1992). Deficiency of acylpeptide hydrolase in small-cell lung carcinoma cell lines. J. Lab. Clin. Med..

[51] Kawahara, M. *et al.* H2.0-like homeobox regulates early hematopoiesis and promotes acute myeloid leukemia. *Cancer Cell* **22**, 194–208. 10.1016/j.ccr.2012.06.027 (2012). 10.1016/j.ccr.2012.06.027 PMC3422691 22897850

[52] Saraiva, D. P. *et al.* Expression of HLA-DR in cytotoxic T lymphocytes: A validated predictive biomarker and a potential therapeutic strategy in breast cancer. *Cancers (Basel)* **13**. 10.3390/cancers13153841 (2021). 10.3390/cancers13153841 PMC8345089 34359741

[53] Thompson L (2006). World Health Organization classification of tumours: Pathology and genetics of head and neck tumours. Ear Nose Throat J..

[54] Chan, A. T. *et al.* Nasopharyngeal cancer: EHNS-ESMO-ESTRO Clinical Practice Guidelines for diagnosis, treatment and follow-up. *Ann. Oncol.* **23**(Suppl 7), vii83–vii85. 10.1093/annonc/mds266 (2012). 10.1093/annonc/mds266 22997460

[55] Huang, S. H. & O'Sullivan, B. Overview of the 8th Edition TNM Classification for Head and Neck Cancer. *Curr. Treat Opt. Oncol.* **18**, 40. 10.1007/s11864-017-0484-y (2017). 10.1007/s11864-017-0484-y 28555375

[56] Tian Y (2017). ChAMP: Updated methylation analysis pipeline for Illumina BeadChips. Bioinformatics.

[57] Pidsley R (2016). Critical evaluation of the Illumina MethylationEPIC BeadChip microarray for whole-genome DNA methylation profiling. Genome Biol..

[58] Wang T (2015). A systematic study of normalization methods for Infinium 450K methylation data using whole-genome bisulfite sequencing data. Epigenetics.

[59] Basu B (2017). Genome-wide DNA methylation profile identified a unique set of differentially methylated immune genes in oral squamous cell carcinoma patients in India. Clin. Epigenet..

[60] Khongsti S (2018). Whole genome DNA methylation profiling of oral cancer in ethnic population of Meghalaya, North East India reveals novel genes. Genomics.

[61] Ernst J, Plasterer HL, Simon I, Bar-Joseph Z (2010). Integrating multiple evidence sources to predict transcription factor binding in the human genome. Genome Res..

[62] Ge SX, Jung D, Yao R (2020). ShinyGO: A graphical gene-set enrichment tool for animals and plants. Bioinformatics.

[63] Zhou G (2019). NetworkAnalyst 3.0: A visual analytics platform for comprehensive gene expression profiling and meta-analysis. Nucleic Acids Res..

[64] Breuer K (2013). InnateDB: Systems biology of innate immunity and beyond—recent updates and continuing curation. Nucleic Acids Res..

