# Supplementary material for: Blood-based DNA methylation in advanced Nasopharyngeal Carcinoma exhibited distinct CpG methylation signature
Source: Sci Rep. 2023 Dec 12;13:22086. doi: 10.1038/s41598-023-45001-w (PMC10716134; doi:10.1038/s41598-023-45001-w)
Supplement: Supplementary file 1 — Supplementary Information. [file 41598_2023_45001_MOESM1_ESM.zip › Supplimentary informations.docx]

**Blood-based DNA methylation in advanced Nasopharyngeal Carcinoma exhibited distinct CpG methylation signature**

Koustav Chatterjee^1^, Sudipa Mal^1^, Monalisha Ghosh^1^, Nabanita Roy Chattopadhyay^1^, Sankar Deb Roy^2^, Koushik Chakraborty^1^, Syamantak Mukherjee^1^, Moatoshi Aier^3^, Tathagata Choudhuri^1*^

^1^Department of Biotechnology, Visva-Bharati, Santiniketan, West Bengal, India.

^2^Department of Radiation Oncology, Eden Medical Center, Dimapur, Nagaland, India.

^3^Department of Pathology, Eden Medical Center, Dimapur, Nagaland, India.

**^*^Correspondence:**

Professor Tathagata Choudhuri. Department of Biotechnology, Visva-Bharati, Santiniketan, Birbhum, West Bengal, India-731235. Email: [tathagata.choudhuri@visva-bharati.ac.in](mailto:tathagata.choudhuri@visva-bharati.ac.in)

**Figure legends:**


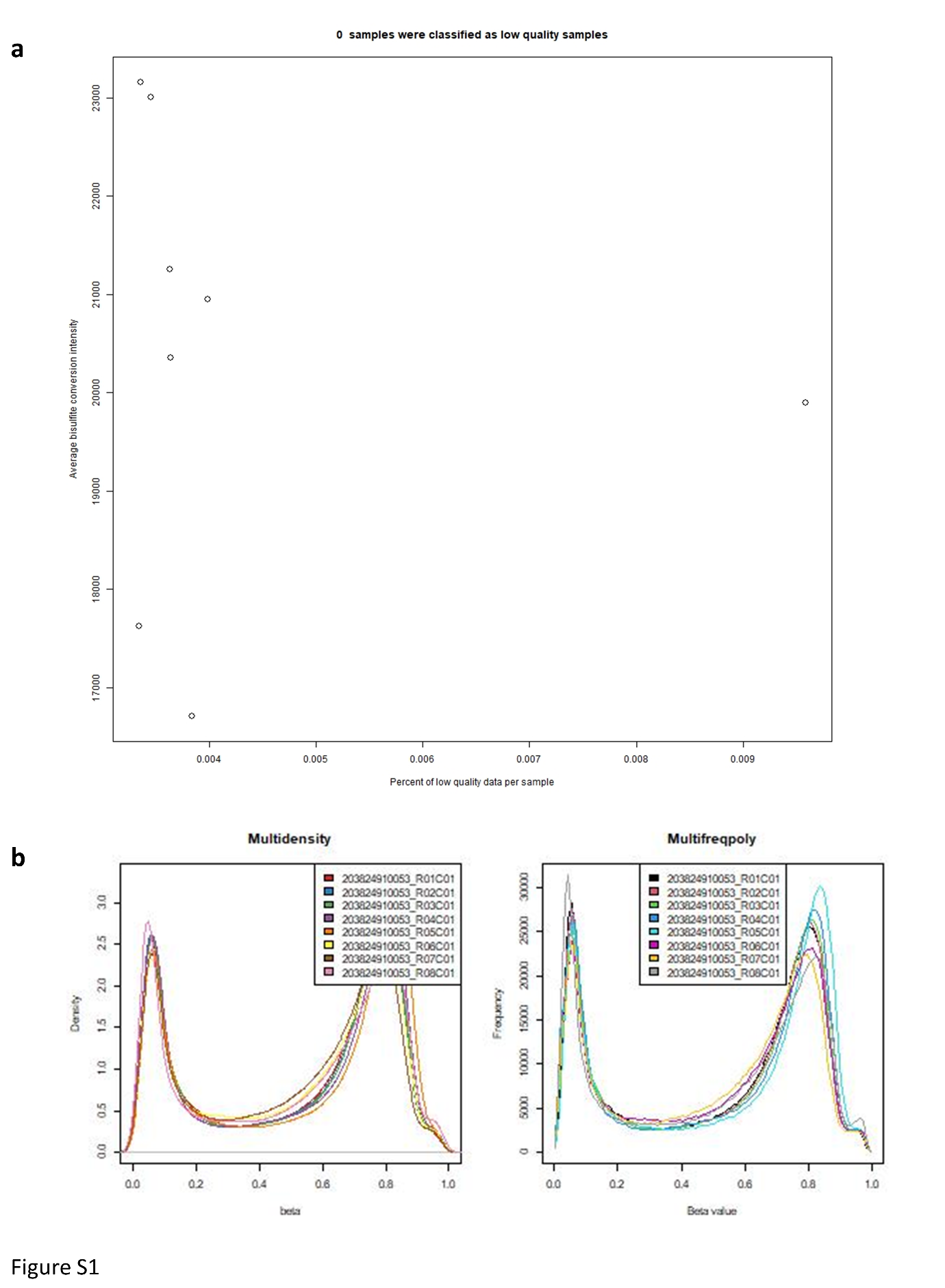


**Figure S1:** QC plots. Panel **a.** measures the Data quality by identifying a list of low-quality samples. Panel **b.** represents the Data distribution plots. Left figure indicates the beta value distribution plots and the right figures shows the Frequency polygon plot, an alternative figure for inspection of data distribution


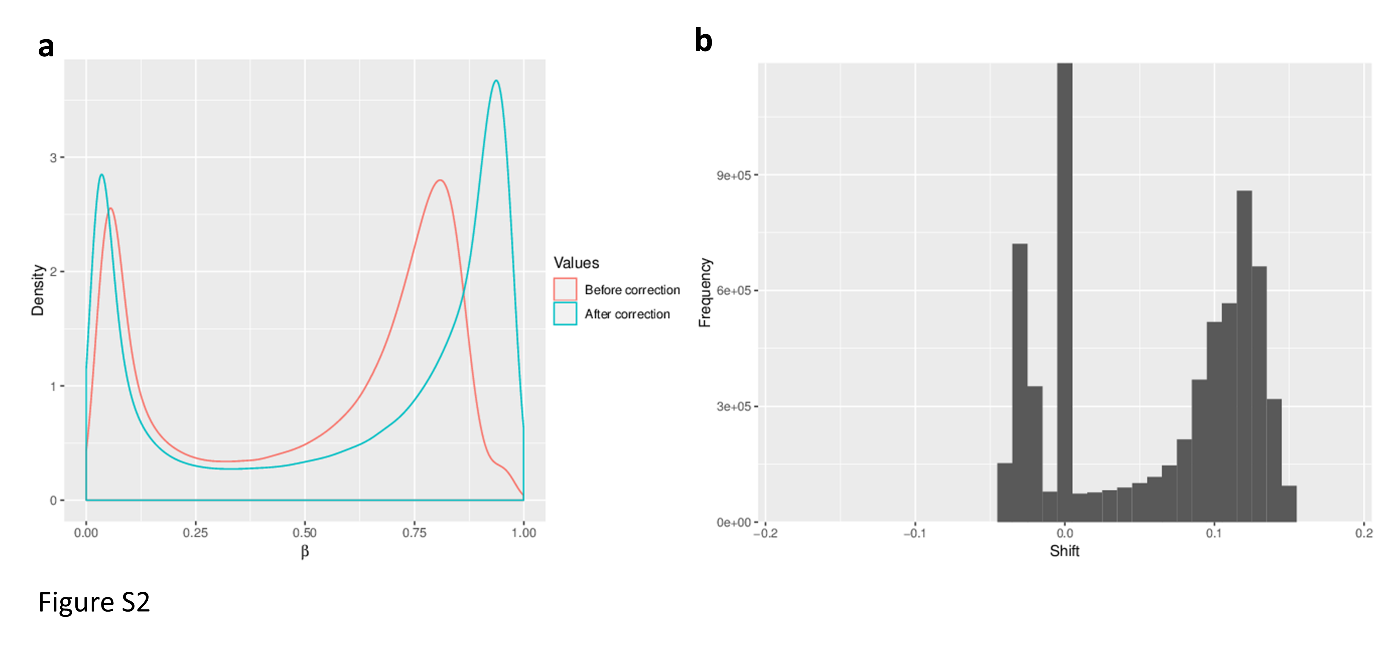


**Figure S2: Normalization of the methyl-array data.** Figure **a.** compares the distributions of the β values before and after performing normalization. Figure **b.** magnitude of the correction by showing the distribution of shifts, i.e., degrees of modification of the raw methylation values.


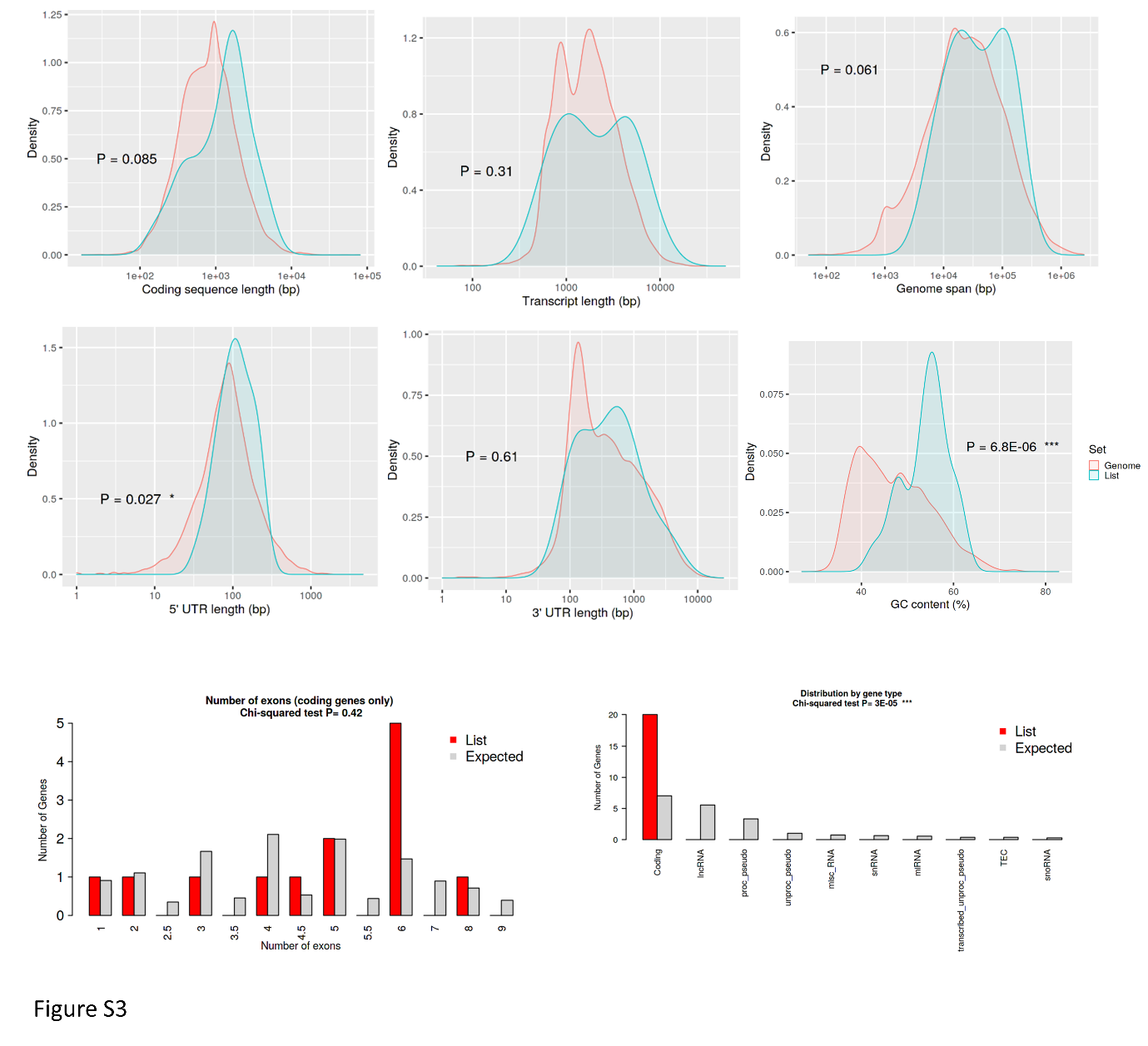


**Figure S3: Characteristics of identified methylated genes.**


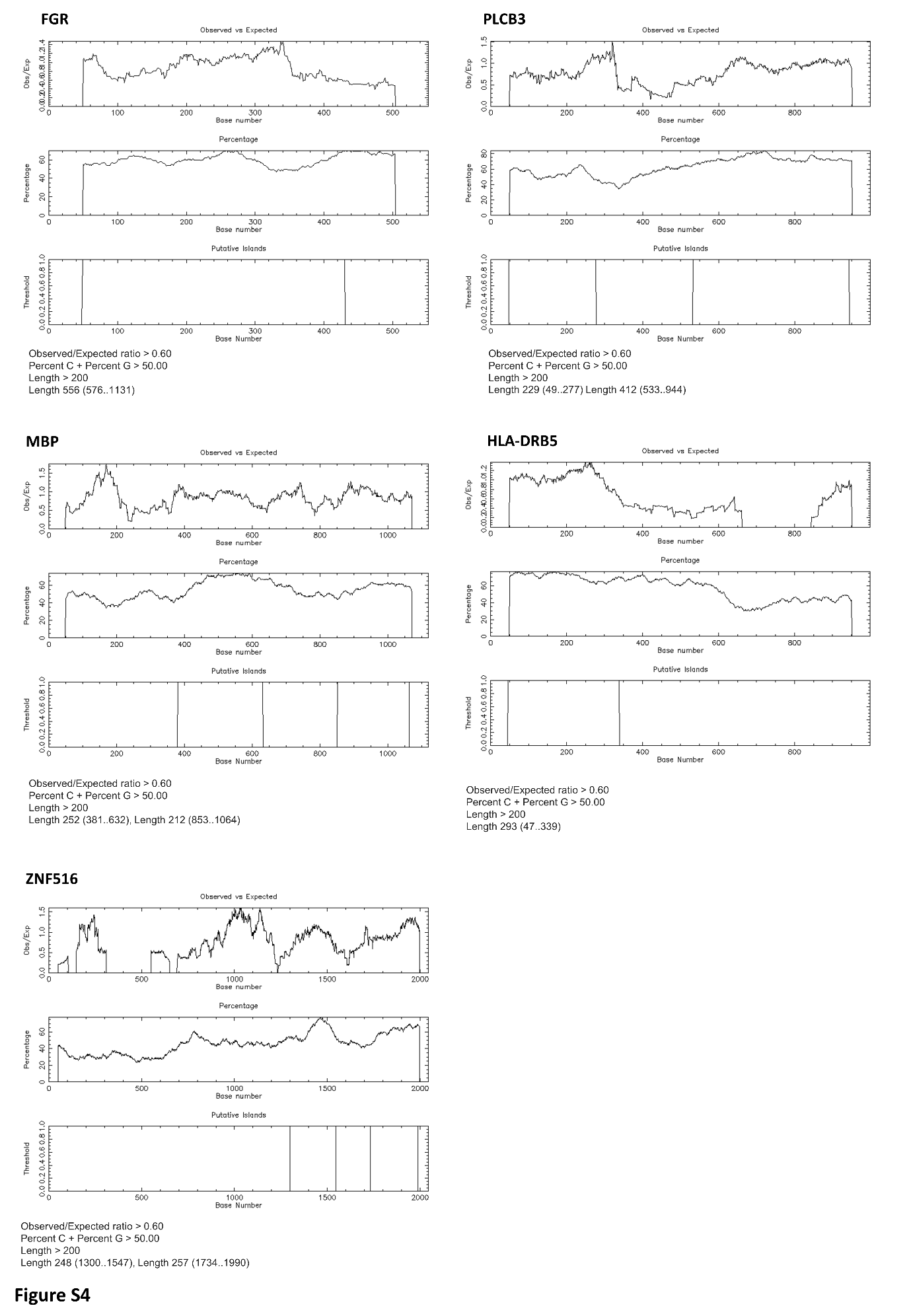


**Figure S4: Identification of CpG island for the selected probes.**


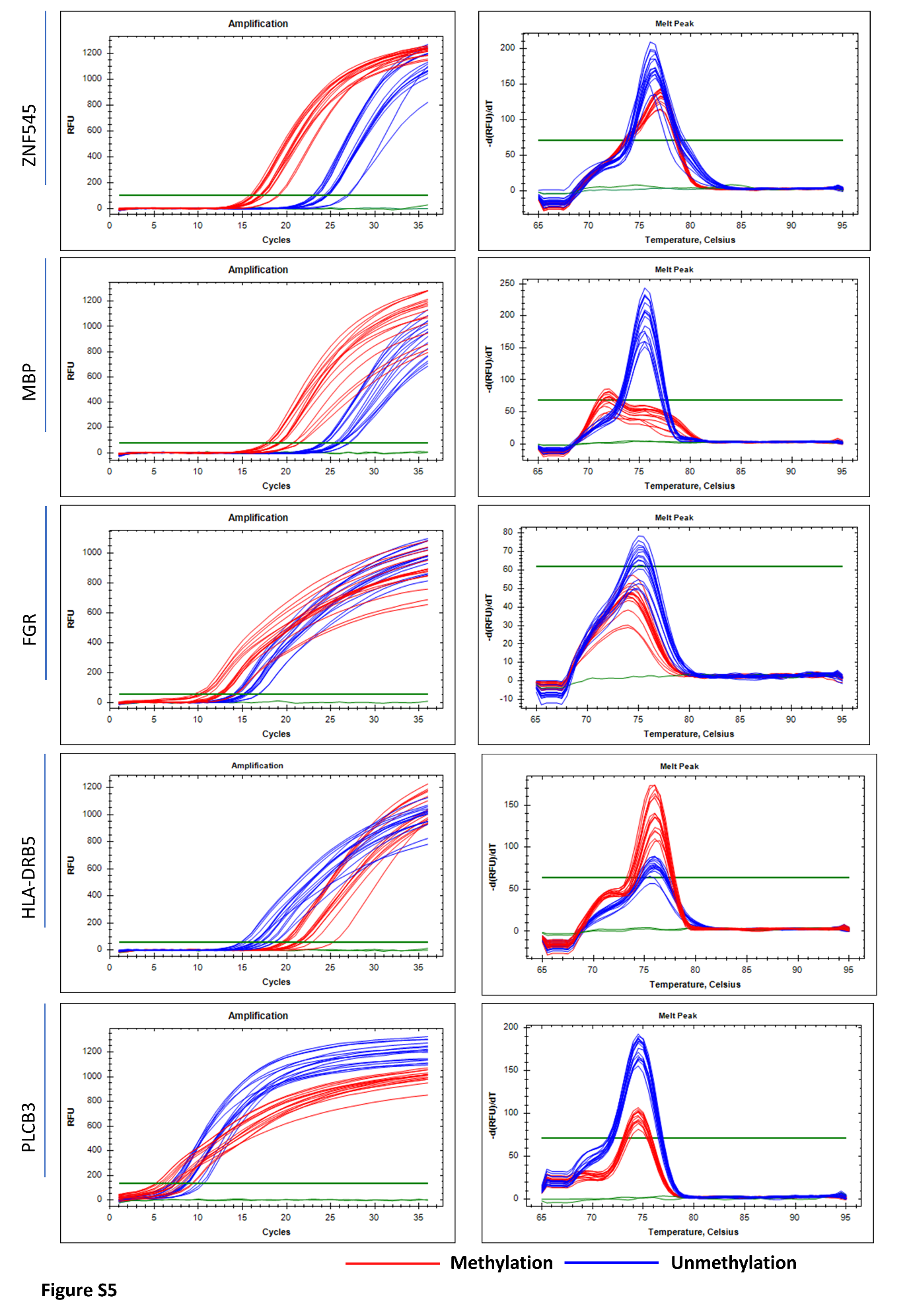


**Figure S5: Amplification and melting curve of the methylated and unmethylated genes in MSP-RT-PCR**

**Table S1: Demographic and Clinical characteristics of NPC cases and healthy controls**

| Sample Group | Age group | Sex | NPC types | Tumor Grade | N | M |
| --- | --- | --- | --- | --- | --- | --- |
| NPC (S1) | 40-50 | M | NKUC | T4 | N3 | Yes |
| NPC (S2) | 50> | M | NKUC | T4 | N4 | Yes |
| NPC (S3) | 50> | M | NKUC | T4 | N4 | Yes |
| NPC (S4) | 40-50 | F | NKUC | T4 | N3 | Yes |
| Control (C1) | 40-50 | M | NA | NA | NA | NA |
| Control (C2) | 40-50 | F | NA | NA | NA | NA |
| Control (C3) | 50> | M | NA | NA | NA | NA |
| Control (C4) | 50> | M | NA | NA | NA | NA |

*Abbreviations: N: tumor spreading to the lymph nodes, M: Metastasis; S: NPC sample, C: Healthy Control*

**Table S2: Differential methylation analysis: please see the Table S2 in .xlsx file.**

**Table S3: Differential hydroxy methylation analysis: please see the Table S3 in .xlsx file.**

**Table S4: determination of corelation between methylation and gene expression through TCGA dataset analysis.**

| Probe ID | **Corelation coefficient (R) and p value (R/P)** | | | | | | | | | | | |
| --- | --- | --- | --- | --- | --- | --- | --- | --- | --- | --- | --- | --- |
|  | BRCA  (n=853) | BLCA  (n=424) | CHOL  (n=45) | COAD  (n=288) | ESCA  (n=190) | HNSC  (n=535) | KIRC  (n=333) | KIRP  (n=292) | LIHC  (n=409) | LUSC  (n=370) | THCA  (n=558) | UCEC  (n=187) |
| cg19006947 | -0.22/  <0.001 | -0.11/  0.02 | -0.05/  0.71 | 0.2/  <0.001 | 0.16/  0.03 | 0.11/  0.01 | 0.073/  0.18 | -0.036/  0.54 | -0.014/  0.78 | -0.28/  <0.001 | -0.18/  <0.001 | -0.089/  0.23 |
| cg12019801 | 0.46/  <0.001 | 0.39/  <0.001 | 0.027/  0.08 | -0.063/  0.29 | 0.31/  <0.001 | 0.11  0.008 | 0.16  0.002 | 0.09/  0.12 | -0.11/  0.03 | 0.18/  <0.001 | 0.01/  0.67 | 0.55/  <0.001 |
| cg16922167 | -0.37/  <0.001 | -0.02/  0.61 | -0.29/  0.05 | 0.28/  <0.001 | 0.024/  0.74 | 0.18/  <0.001 | 0.087/  0.11 | -0.32/  <0.001 | -0.24/  <0.001 | -0.2/  0.00014 | -0.15/  0.00028 | -0.019/  0.79 |
| cg18375707 | -0.22/  <0.001 | -0.12/  0.012 | 0.21/  0.16 | -0.22/  0.00022 | -0.26/  0.00024 | 0.033/  0.44 | 0.058/  0.29 | 0.011/  0.85 | 0.044/  0.38 | -0.32/  <0.001 | -0.16/  0.00022 | -0.064/  0.39 |
| cg26930596 | <0.001/  0.99 | 0.34/  <0.001 | -0.34/  0.023 | -0.085/  0.15 | -0.4/  <0.001 | -0.096/  0.027 | 0.2/  0.00025 | 0.2/  0.00055 | 0.2/  <0.001 | -0.17/  0.00082 | -0.01/  0.81 | -0.03/  0.68 |
| cg15621731 | 0.26/  <0.001 | 0.29/  <0.001 | -0.1/  0.5 | -0.042/  0.48 | 0.14/  0.055 | 0.21/  <0.001 | 0.085/  0.12 | 0.26/  <0.001 | -0.045/  0.37 | 0.19/  0.00033 | 0.32/  <0.001 | -0.16/  0.024 |
| cg09255910 | -0.031/  0.37 | -0.069/  0.16 | -0.13/  0.41 | -0.52/  <0.001 | -0.095/  0.19 | -0.0032/  0.94 | 0.065/  0.24 | -0.21/  0.00037 | 0.0048/  0.92 | -0.16/  0.0019 | -0.14/  0.00062 | -0.25/  0.00062 |
| cg00471371 | 0.29/  <0.001 | 0.26/  <0.001 | 0.047/  0.76 | -0.04/  0.5 | 0.3/  <0.001 | 0.082/  0.058 | 0.14/  0.011 | 0.086/  0.14 | -0.047/  0.35 | 0.15/  0.0043 | -0.075/  0.078 | -0.57/  <0.001 |
| cg01662869 | -0.27/  <0.001 | -0.096/  0.049 | 0.22/  0.15 | -0.13/  0.034 | -0.32/  <0.001 | 0.062/  0.15 | -0.23/  <0.001 | -0.02/  0.73 | -0.028/  0.57 | -0.085/  0.1 | -0.29/  <0.001 | -0.082/  0.27 |
| cg18351781 | -0.044/  0.2 | -0.081/  0.095 | -0.27/  0.069 | -0087/  0.14 | 0.082/  0.26 | 0.038/  0.38 | -0.31/  <0.001 | -0.017/  0.78 | -0.11/  0.033 | 0.24/  <0.001 | -0.26/  <0.001 | -0.12/  0.093 |
| cg15982099 | -0.58/  <0.001 | -0.63/  <0.001 | -0.59/  <0.001 | -0.53/  <0.001 | -0.31/  <0.001 | -0.063/  0.15 | -0.54/  <0.001 | -0.75/  <0.001 | -0.64/  <0.001 | -0.19/  0.00018 | -0.69/  <0.001 | -0.21/  0.0042 |
| cg02668773 | -0.23/  <0.001 | -0.021/  0.67 | -0.3/  0.048 | -0.29  <0.001 | -0.0023/  0.98 | -0.053/  0.22 | 0.019/  0.72 | -0.011/  0.85 | -0.019/  0.7 | -0.056/  0.28 | -0.025/  0.56 | -0.28/  0.00014 |
| cg08670658 | -0.18/  <0.001 | 0.052/  0.28 | -0.011/  0.94 | -0.026/  0.66 | -0.089/  0.22 | 0.067/  0.12 | -0.018/  0.74 | -0.049/  0.41 | -0.071/  0.15 | 0.071/  0.17 | -0.04/  0.34 | 0.0035/  0.96 |
| cg02266086 | -0.49/  <0.001 | -0.041/  0.4 | -0.11/  0.47 | -0.21/  0.00038 | -0.33/  <0.001 | -0.33/  <0.001 | -0.15/  0.0068 | -0.042/  0.48 | -0.21/  <0.001 | -0.33/  <0.001 | 0.025/  0.56 | -0.3/  <0.001 |
| cg06633438 | -0.0052/  0.88 | -0.1/  0.035 | 0.42/  0.0045 | -0.03/  0.61 | 0.19/  0.0086 | 0.077/  0.075 | -0.17/  0.0018 | -0.12/  0.035 | 0.044/  0.38 | 0.012/  0.81 | -0.24/  <0.001 | -0.057/  0.44 |
| cg10283505 | -0.33/  <0.001 | -0.38/  <0.001 | -0.67/  <0.001 | -0.28/  <0.001 | -0.65/  <0.001 | -0.21/  <0.001 | -0.18/  0.0012 | -0.34/  <0.001 | 0.29/  <0.001 | -0.39/  <0.001 | -0.25/  <0.001 | -0.43/  <0.001 |
| cg25389087 | 0.16/  <0.001 | -0.093/  0.055 | <0.001/  1 | -0.17/  0.0031 | 0.18/  0.014 | 0.011/  0.8 | 0.18/  0.00086 | -0.051/  0.39 | -0.13/  0.0088 | 0.027/  0.61 | -0.07/  0.099 | 0.11/  0.12 |
| cg23730027 | -0.87/  <0.001 | -0.24/  <0.001 | -0.034/  0.83 | 0.12/  0.041 | -0.2/  0.0059 | -0.18/  <0.001 | -0.29/  <0.001 | -0.38  <0.001 | -0.29/  <0.001 | -0.26/  <0.001 | -0.38/  <0.001 | -0.28/  <0.001 |
| cg01546248 | -0.073/  0.14 | -0.073/  0.14 | -0.015/  0.92 | -0.06/  0.33 | 0.007/  0.93 | -0.022/  0.61 | 0.15/  0.0049 | -0.037/  0.54 | 0.0049/  0.93 | -0.0095/  0.86 | 0.002/  0.96 | -0.027/  0.72 |
| cg01978534 | -0.055/  0.11 | -0.19/  <0.001 | -0.17/  0.26 | -0.049/  0.41 | -0.18/  0.014 | -0.073/  0.094 | -0.13/  0.016 | -0.18/  0.002 | -0.16/  0.001 | -0.077/  0.14 | 0.0025/  0.95 | -0.08/  0.28 |
| cg26981746 | 0.081/  0.073 | 0.44/  <0.001 | -0.11/  0.65 | 0.21/  0.013 | 0.38/  <0.001 | 0.43/  <0.001 | 0.037/  0.64 | 0.22/  0.0042 | 0.17/  0.0096 | 0.38/  <0.001 | -0.041/  0.5 | 0.38/  <0.001 |

BLCA: bladder urothelial carcinoma, BRCA: breast invasive carcinoma, CHOL: cholangio carcinoma, COAD: colon adenocarcinoma, ESCA: esophageal carcinoma, HNSC: head and neck squamous cell carcinoma, KIRC: kidney renal clear cell carcinoma, KIRP: kidney renal papillary cell carcinoma, LIHC: liver hepatocellular carcinoma, LUSC: lung squamous cell carcinoma, THCA: thyroid carcinoma, UCEC: uterine corpus endometrial carcinoma (UCEC)

P value ≤ 0.05 was considered as statistically significant.

**Table S5: Primer sequences for MSP-RT-PCR**

| **Genes** |  | **Tm** | **%GC** | **C's** | **Sequence 5'-3'** |
| --- | --- | --- | --- | --- | --- |
| **FGR** | Forward M | 58.99 | 52 | 5 | TTTTTAAAGTGTTGGGATTATAGGC |
|  | Reverse M | 59.76 | 68.18 | 8 | AAAAATACCCGAAAAACACGAA |
|  | Forward U | 59.96 | 55.56 | 6 | TTTTTTAAAGTGTTGGGATTATAGGTG |
|  | Reverse U | 55.56 | 68.18 | 8 | AAAAATACCCAAAAAACACAAA |
| **PLCB3** | Forward M | 59.64 | 64 | 6 | ATTTAGGTTGGAGTGTAATGGTACG |
|  | Reverse M | 59.68 | 60 | 9 | AATAAATCACGAAATCAAAAATTCG |
|  | Forward U | 58.91 | 64 | 6 | TTTAGGTTGGAGTGTAATGGTATGA |
|  | Reverse U | 57.06 | 56 | 8 | TAAATCACAAAATCAAAAATTCAAA |
| **MBP** | Forward M | 59.24 | 42.31 | 4 | TAGTTGAAAGTTATTTTTCGAAATCG |
|  | Reverse M | 59.18 | 60.87 | 5 | GAAAAACCTACGTTAACAAACGC |
|  | Forward U | 58.38 | 44.44 | 4 | TAGTTGAAAGTTATTTTTTGAAATTGG |
|  | Reverse U | 57.43 | 60 | 5 | CAAAAAACCTACATTAACAAACACA |
| **ZNF516** | Forward M | 59.11 | 68 | 6 | ACGAGGTCGTTGTAAAGTAGGTATC |
|  | Reverse M | 59.3 | 63.64 | 4 | CTAAAAACTTCCGAACCTCGAC |
|  | Forward U | 57.07 | 69.23 | 6 | ATGAGGTTGTTGTAAAGTAGGTATTG |
|  | Reverse U | 58.88 | 60 | 4 | CTTCTAAAAACTTCCAAACCTCAAC |
| **HLA-DRB5** | Forward M | 57.77 | 60.87 | 4 | CGTAGAGATATATGTTAGCGCGT |
|  | Reverse M | 59.07 | 52 | 4 | TCGAATTACTAAAATTCTACCCGTC |
|  | Forward U | 52.8 | 62.96 | 5 | TAGGTGTAGAGATATATGTTAGTGTGT |
|  | Reverse U | 55.12 | 52 | 4 | TCAAATTACTAAAATTCTACCCATC |

Table S6: **Primer sequences for quantitative RT-PCR**

| Gene | Direction | Sequences 5'-3' | TM | GC | Self-complementarity | Self3' complementarity |
| --- | --- | --- | --- | --- | --- | --- |
| FGR | Forward | GGCTAGGGTGGAGACCAAAG | 59.75 | 60 | 4 | 2 |
|  | Reverse | TTCCCGAATGAGAAAGGCCC | 60.03 | 55 | 4 | 3 |
| PLCB3 | Forward | AGAACAGACAGGTGCAGAGC | 59.97 | 55 | 4 | 2 |
|  | Reverse | AAGACTGAGGGCAGGAAGGA | 60.18 | 55 | 3 | 0 |
| MBP | Forward | GGATCACCCATGGCTAGACG | 59.97 | 60 | 6 | 2 |
|  | Reverse | TCTGTCTCTGCAGCTGTGTG | 59.97 | 55 | 6 | 2 |
| HLA-DRB5 | Forward | ACCCAACAGTGCTCTCATCTG | 60 | 52.38 | 4 | 2 |
|  | Reverse | GGAAGCCACAAGGATGGACA | 59.96 | 55 | 3 | 2 |
| ZNF516 | Forward | CGGGCGCTCGGCTTC | 60.65 | 80 | 6 | 0 |
|  | Reverse | CAATGACAACGCTCCCAGTT | 58.76 | 50 | 3 | 2 |

**Table S7: Analysis of transcription factor binding sites analysis in methylated CpG**

| **Gene** | **Chr** | **Chr. Start** | **Chr. End** | **Matrix name** | **Score** | **Strand** | **TF Name** |
| --- | --- | --- | --- | --- | --- | --- | --- |
| PRKCZ | 1 | 2082393 | 2082409 | MA0041.2 | 627 | - | FOXD3 |
| FGR | 1 | 27961715 | 27961728 | MA0697.2 | 644 | - | Zic3 |
| KDM4B | 19 | 5074789 | 5074801 | MA1619.1 | 674 | + | Ptf1A |
| HLX | 1 | 221055592 | 2.21E+08 | MA0152.2 | 644 | + | Nfatc2 |
| MPO | 17 | 56356707 | 56356721 | MA1784.1 | 624 | - | FOXO1 |
|  | 17 | 56356836 | 56356849 | MA0698.1 | 734 | + | ZBTB18 |
| COL11A2 | 6 | 33161209 | 33161220 | MA2047.1 | 614 | - | Nr1H2 |
| MLLT1 | 19 | 6272083 | 6272095 | MA1866.1 | 611 | - | PATZ1 |
| FUT4 | 11 | 94277051 | 94277072 | MA1418.1 | 611 | + | IRF3 |
|  | 11 | 94277058 | 94277071 | MA1623.1 | 650 | + | Stat2 |
|  | 11 | 94277074 | 94277084 | MA1562.1 | 602 | + | SOX14 |
|  | 11 | 94277769 | 94277782 | MA0800.1 | 734 | - | EOMES |
|  | 11 | 94277853 | 94277865 | MA1707.1 | 602 | + | DMRTA1 |
|  | 11 | 94278393 | 94278404 | MA0480.2 | 662 | + | Foxo1 |
|  | 11 | 94278523 | 94278538 | MA1729.1 | 603 | + | ZNF680 |
|  | 11 | 94278803 | 94278816 | MA0697.2 | 712 | - | Zic3 |
|  | 11 | 94278805 | 94278816 | MA1628.1 | 602 | - | Zic1 |
|  | 11 | 94278814 | 94278831 | MA0731.1 | 619 | - | BCL6B |
| FLNB | 3 | 57994952 | 57994966 | MA1587.1 | 603 | - | ZNF135 |
|  | 3 | 57995090 | 57995106 | MA1596.1 | 963 | - | ZNF460 |
|  | 3 | 57995201 | 57995217 | MA0041.2 | 614 | + | FOXD3 |
| SMTN | 22 | 31476889 | 31476905 | MA1596.1 | 863 | - | ZNF460 |
|  | 22 | 31476980 | 31476992 | MA1125.1 | 622 | + | ZNF384 |
|  | 22 | 31477147 | 31477162 | MA0728.1 | 622 | + | Nr2F6 |
|  | 22 | 31477147 | 31477163 | MA0859.1 | 634 | + | Rarg |
| APEH | 3 | 49711119 | 49711135 | MA0859.1 | 634 | + | Rarg |
|  | 3 | 49711797 | 49711810 | MA0017.2 | 620 | - | NR2F1 |
|  | 3 | 49711995 | 49712019 | MA2033.1 | 626 | - | THRA |
|  | 3 | 49712008 | 49712023 | MA0728.1 | 622 | + | Nr2F6 |
|  | 3 | 49712114 | 49712130 | MA1596.1 | 963 | - | ZNF460 |
|  | 3 | 49712213 | 49712225 | MA1125.1 | 611 | + | ZNF384 |
| HLA-DRB5 | 6 | 32490016 | 32490037 | MA1872.1 | 617 | + | ZNF354A |

Abbreviations: TF: Transcription factor, Chr: Chromosome no, (+) ve: sense strand, (-)ve: antisense strand

**Table S8: Result of gene enrichment analysis**

| **Enrichment FDR** | **Fold Enrichment** | **Pathway** | **URL** | **Gene involve** |
| --- | --- | --- | --- | --- |
| 0.016553018 | 8.074144038 | Cell activation involved in immune response | GO:0002263 | FGR MPO SPI1 PRKCZ HLX APEH |
| 0.016553018 | 8.112455516 | Leukocyte activation involved in immune response | GO:0002366 | FGR MPO SPI1 PRKCZ HLX APEH |
| 0.016553018 | 10.65233645 | Regulation of cell-cell adhesion | GO:0022407 | SPI1 PRKCZ HLX FUT4 MBP |
| 0.016553018 | 151.9733333 | T-helper 2 cell differentiation | GO:0045064 | PRKCZ HLX |
| 0.016553018 | 207.2363636 | Regulation of T-helper 2 cell differentiation | GO:0045628 | PRKCZ HLX |
| 0.019024264 | 15.50748299 | Positive regulation of immune effector process | GO:0002699 | FGR SPI1 PRKCZ HLX |
| 0.022219706 | 108.552381 | Positive regulation of T-helper cell differentiation | GO:0045624 | PRKCZ HLX |
| 0.041389753 | 4.124728589 | Cell activation | GO:0001775 | FGR MPO SPI1 PRKCZ HLX APEH |
| 0.041389753 | 5.493012048 | Immune effector process | GO:0002252 | FGR MPO SPI1 PRKCZ HLX APEH |
| 0.041389753 | 6.992638037 | Myeloid cell activation involved in immune response | GO:0002275 | FGR MPO SPI1 APEH |
| 0.041389753 | 7.806849315 | Neutrophil activation involved in immune response | GO:0002283 | FGR MPO SPI1 APEH |
| 0.041389753 | 16.05352113 | Lymphocyte activation involved in immune response | GO:0002285 | SPI1 PRKCZ HLX |
| 0.041389753 | 30.80540541 | Alpha-beta T cell activation involved in immune response | GO:0002287 | PRKCZ HLX |
| 0.041389753 | 28.495 | T cell differentiation involved in immune response | GO:0002292 | PRKCZ HLX |
| 0.041389753 | 30.80540541 | Alpha-beta T cell differentiation involved in immune response | GO:0002293 | PRKCZ HLX |
| 0.041389753 | 31.22739726 | CD4-positive, alpha-beta T cell differentiation involved in immune response | GO:0002294 | PRKCZ HLX |
| 0.041389753 | 5.845128205 | Leukocyte mediated immunity | GO:0002443 | FGR MPO SPI1 PRKCZ APEH |
| 0.041389753 | 6.907878788 | Myeloid leukocyte mediated immunity | GO:0002444 | FGR MPO SPI1 APEH |
| 0.041389753 | 7.68836425 | Neutrophil mediated immunity | GO:0002446 | FGR MPO SPI1 APEH |
| 0.041389753 | 379.9333333 | Pro-T cell differentiation | GO:0002572 | SPI1 |
| 0.041389753 | 7.248330684 | Regulation of leukocyte activation | GO:0002694 | FGR SPI1 PRKCZ HLX |
| 0.041389753 | 15.26517857 | Negative regulation of leukocyte activation | GO:0002695 | FGR SPI1 HLX |
| 0.041389753 | 9.825862069 | Regulation of immune effector process | GO:0002697 | FGR SPI1 PRKCZ HLX |
| 0.041389753 | 12.08268551 | Regulation of leukocyte mediated immunity | GO:0002703 | FGR SPI1 PRKCZ |
| 0.041389753 | 71.2375 | Regulation of type 2 immune response | GO:0002828 | PRKCZ HLX |
| 0.041389753 | 37.3704918 | Regulation of myeloid leukocyte mediated immunity | GO:0002886 | FGR SPI1 |
| 0.041389753 | 31.22739726 | Protein monoubiquitination | GO:0006513 | MGRN1 UHRF1 |
| 0.041389753 | 33.52352941 | Response to fungus | GO:0009620 | MPO SPI1 |
| 0.041389753 | 15.4027027 | Negative regulation of cell-cell adhesion | GO:0022408 | SPI1 HLX MBP |
| 0.041389753 | 6.808841099 | Regulation of cell adhesion | GO:0030155 | SPI1 PRKCZ HLX FUT4 MBP |
| 0.041389753 | 12.16868327 | T cell differentiation | GO:0030217 | SPI1 PRKCZ HLX |
| 0.041389753 | 46.52244898 | Negative regulation of histone modification | GO:0031057 | SPI1 KDM4B |
| 0.041389753 | 7.64966443 | Negative regulation of protein modification process | GO:0031400 | SPI1 PRKCZ KDM4B MLLT1 |
| 0.041389753 | 7.523432343 | Granulocyte activation | GO:0036230 | FGR MPO SPI1 APEH |
| 0.041389753 | 63.32222222 | Type 2 immune response | GO:0042092 | PRKCZ HLX |
| 0.041389753 | 32.10704225 | T-helper cell differentiation | GO:0042093 | PRKCZ HLX |
| 0.041389753 | 7.636850921 | Neutrophil activation | GO:0042119 | FGR MPO SPI1 APEH |
| 0.041389753 | 7.134898279 | Leukocyte degranulation | GO:0043299 | FGR MPO SPI1 APEH |
| 0.041389753 | 44.69803922 | Regulation of leukocyte degranulation | GO:0043300 | FGR SPI1 |
| 0.041389753 | 7.915277778 | Neutrophil degranulation | GO:0043312 | FGR MPO SPI1 APEH |
| 0.041389753 | 40.70714286 | Regulation of CD4-positive, alpha-beta T cell differentiation | GO:0043370 | PRKCZ HLX |
| 0.041389753 | 61.61081081 | Positive regulation of CD4-positive, alpha-beta T cell differentiation | GO:0043372 | PRKCZ HLX |
| 0.041389753 | 379.9333333 | Histone H3-K27 acetylation | GO:0043974 | SPI1 |
| 0.041389753 | 379.9333333 | DNA hypermethylation | GO:0044026 | SPI1 |
| 0.041389753 | 4.636474576 | Leukocyte activation | GO:0045321 | FGR MPO SPI1 PRKCZ HLX APEH |
| 0.041389753 | 51.80909091 | Regulation of T-helper cell differentiation | GO:0045622 | PRKCZ HLX |
| 0.041389753 | 36.18412698 | Negative regulation of G protein-coupled receptor signaling pathway | GO:0045744 | MGRN1 GRK2 |
| 0.041389753 | 28.14320988 | Positive regulation of alpha-beta T cell activation | GO:0046635 | PRKCZ HLX |
| 0.041389753 | 30.80540541 | Regulation of alpha-beta T cell differentiation | GO:0046637 | PRKCZ HLX |
| 0.041389753 | 40.70714286 | Positive regulation of alpha-beta T cell differentiation | GO:0046638 | PRKCZ HLX |
| 0.041389753 | 6.86626506 | Positive regulation of immune response | GO:0050778 | FGR SPI1 PRKCZ HLX HLA-DRB5 |
| 0.041389753 | 42.21481481 | Defense response to fungus | GO:0050832 | MPO SPI1 |
| 0.041389753 | 6.724483776 | Regulation of cell activation | GO:0050865 | FGR SPI1 PRKCZ HLX |
| 0.041389753 | 13.95673469 | Negative regulation of cell activation | GO:0050866 | FGR SPI1 HLX |
| 0.041389753 | 55.6 | Adipose tissue development | GO:0060612 | SPI1 ZNF516 |
| 0.041389753 | 13.00152091 | Connective tissue development | GO:0061448 | SPI1 ZNF516 COL11A2 |
| 0.041389753 | 379.9333333 | Oncogene-induced cell senescence | GO:0090402 | SPI1 |
| 0.041389753 | 379.9333333 | Endothelial to hematopoietic transition | GO:0098508 | SPI1 |
| 0.041389753 | 5.699 | Cell-cell adhesion | GO:0098609 | SPI1 PRKCZ HLX FUT4 MBP |
| 0.041389753 | 379.9333333 | Regulation of histone H3-K27 acetylation | GO:1901674 | SPI1 |
| 0.041389753 | 11.13811075 | Positive regulation of leukocyte cell-cell adhesion | GO:1903039 | PRKCZ HLX FUT4 |
| 0.041389753 | 379.9333333 | Negative regulation of adipose tissue development | GO:1904178 | SPI1 |
| 0.041389753 | 379.9333333 | Regulation of antifungal innate immune response | GO:1905034 | SPI1 |
| 0.041389753 | 379.9333333 | Regulation of myeloid progenitor cell differentiation | GO:1905453 | SPI1 |
| 0.041389753 | 50.65777778 | Positive regulation of CD4-positive, alpha-beta T cell activation | GO:2000516 | PRKCZ HLX |
| 0.042378039 | 10.52123077 | Negative regulation of cell adhesion | GO:0007162 | SPI1 HLX MBP |
| 0.042696536 | 25.32888889 | CD4-positive, alpha-beta T cell differentiation | GO:0043367 | PRKCZ HLX |
| 0.043923729 | 24.77826087 | Regulation of CD4-positive, alpha-beta T cell activation | GO:2000514 | PRKCZ HLX |
| 0.045384809 | 4.78907563 | Positive regulation of immune system process | GO:0002684 | FGR SPI1 PRKCZ HLX HLA-DRB5 |
| 0.045384809 | 10.05705882 | Muscle organ development | GO:0007517 | FLNB HLX SMTN |
| 0.04538756 | 284.95 | Response to yeast | GO:0001878 | MPO |
| 0.04538756 | 284.95 | Follicular B cell differentiation | GO:0002316 | SPI1 |
| 0.04538756 | 284.95 | Myeloid dendritic cell chemotaxis | GO:0002408 | SPI1 |
| 0.04538756 | 284.95 | Negative regulation of T-helper 2 cell differentiation | GO:0045629 | HLX |
| 0.04538756 | 284.95 | Microglial cell mediated cytotoxicity | GO:0090634 | SPI1 |
| 0.04538756 | 284.95 | Regulation of defense response to fungus | GO:1900150 | SPI1 |
| 0.04538756 | 284.95 | Negative regulation of neutrophil activation | GO:1902564 | SPI1 |
| 0.04538756 | 284.95 | Regulation of microglial cell mediated cytotoxicity | GO:1904149 | SPI1 |
| 0.04538756 | 284.95 | Positive regulation of microglial cell mediated cytotoxicity | GO:1904151 | SPI1 |
| 0.045915157 | 9.551396648 | Positive regulation of cell-cell adhesion | GO:0022409 | PRKCZ HLX FUT4 |
| 0.049742049 | 9.241621622 | Negative regulation of immune response | GO:0050777 | FGR SPI1 HLX |
| 0.049856921 | 21.10740741 | Positive regulation of T cell differentiation | GO:0045582 | PRKCZ HLX |
